# Orbital Interactions in Hydrogen Bonds: A Perspective From the Chemical Bond Overlap Model

**DOI:** 10.1002/jcc.70166

**Published:** 2025-07-10

**Authors:** Rodolfo A. Santos, Carlos V. Santos‐, Eduardo C. Aguiar, Albano N. Carneiro Neto, Renaldo T. Moura

**Affiliations:** ^1^ Department of Chemistry Federal University of Paraiba João Pessoa Brazil; ^2^ Academic Unit of Belo Jardim Federal Rural University of Pernambuco Belo Jardim Brazil; ^3^ Physics Department University of Aveiro Aveiro Portugal; ^4^ Computational and Theoretical Chemistry Group (CATCO), Department of Chemistry Southern Methodist University Dallas Texas USA; ^5^ Academic Unit of Cabo de Santo Agostinho Federal Rural University of Pernambuco Cabo de Santo Agostinho Brazil

**Keywords:** electron density, hydrogen bonding, OP/TOP models, orbital overlap, QTAIM

## Abstract

Hydrogen bonds are essential chemical interactions that occur in various systems, playing a critical role in determining molecular structures, dynamics, and reactivity. While quantum chemical methods such as Quantum Theory of Atoms in Molecules (QTAIM) and Natural Bond Orbital (NBO) analyses have traditionally been used to explore these interactions, this work introduces the Chemical Bond Overlap (OP) Model and its topological (TOP) descriptors as a complementary approach for analyzing orbital overlap contributions in hydrogen bonds. The study reports a systematic investigation of a series of hydrogen‐bonded systems (a total of 25 systems), demonstrating how electron‐donating and electron‐withdrawing substituents influence bond characteristics. The results reveal that OP/TOP effectively captures the effects of electronic perturbations, offering insights into the n(X) → $\sigma^{*}({\text{X}}^{\prime }-\text{H})$ interactions and serving as a complementary approach to QTAIM, NBO, and local vibrational modes theory (LVM). Notably, for nonconventional ${\left({\text{CH}}_{3}\right)}_{3}\text{N}\text{⋯}\text{H}\text{⋯}{\text{CX}}_{3}$ hydrogen bonds (X=F,Cl), the OP/TOP model, consistent with LVM, correctly captures the expected increase in interaction strength for X=Cl. This agrees with the higher electrophilicity of the hydrogen in ${\text{HCCl}}_{3}$, as indicated by its lower pKa and weaker CH bond dissociation energy. Additionally, the inclusion of electron‐donating groups significantly enhances lone pair → antibonding orbital interactions, increasing NBO $\sigma^{*}({\text{X}}^{\prime }-\text{H})$ occupancy and electron density at the hydrogen bond critical point (BCP), as reflected by a decrease in ${\text{H}}_{\text{BCP}}$ and an increase in $\rho_{\text{BCP}}$. This behavior consistently indicates hydrogen bond strengthening across QTAIM, NBO, and OP/TOP descriptors. Calculations were performed using the ωB97X‐D/def2‐TZVP level of theory. The findings establish OP/TOP as a powerful tool for computational chemistry, particularly in the study of weak intermolecular interactions and molecular design.

## Graphical abstract

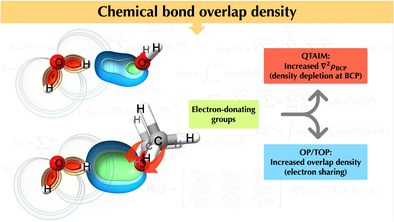

## 1 Introduction

The hydrogen bond (HB) is an important type of interaction that arises in either intermolecular or intramolecular cases. It involves a donor group (HB donor) that contains a hydrogen atom bound to a molecular fragment. The interaction also includes an electron‐rich region, called the acceptor group (HB acceptor). The acceptor is usually an electronegative atom with a lone pair or a π‐bond. Due to its complex nature, the definition of the HB has been refined by IUPAC through the consideration of multiple criteria [1]. Since its initial description by Moore and Winmill in 1912 [2, 3], hydrogen bonding and its associated interactions have been extensively investigated across diverse chemical environments, from both experimental and theoretical perspectives.

Several quantum chemical models have been employed to elucidate the nature of hydrogen bonds. These include energy decomposition analysis (EDA) [4, 5, 6, 7, 8, 9, 10, 11], topological analysis of electron density via quantum theory of atoms in molecules (QTAIM) [12, 13, 14, 15, 16, 17, 18, 19, 20, 21, 22, 23, 24, 25, 26, 27, 28, 29, 30, 31], electron localization function (ELF) analysis [32, 33, 34, 35, 36], natural bond orbital (NBO) theory [37, 38, 39, 40, 41, 42], and local vibrational mode (LVM) theory [43, 44, 45, 46, 47, 48], among the most widely used approaches. Notable contributions include studies by Gadre and collaborators [49, 50, 51, 52, 53, 54] on hydrogen bond energetics and by Suresh and colleagues [55, 56, 57, 58] on the application of electrostatic potential in hydrogen bonding.

According to NBO analysis, the main interaction in HB formation, ${\text{X}}^{\prime }-\text{H}\text{⋯}\text{X}-\text{R}$, is a stabilizing charge transfer. It occurs from the lone pair on X to the antibonding orbital of ${\text{X}}^{\prime }-\text{H}$, $\text{n}(\text{X})\to \sigma^{*}{\text{X}}^{\prime }-\text{H}$ [59]. Alabugin and collaborators [60, 61] point out that this hyperconjugative interaction is counterbalanced by rehybridization. This effect involves increased s‐character on ${\text{X}}^{\prime }$ and polarization of the ${\text{X}}^{\prime }-\text{H}$ bond. Similar findings are reported in other studies [62].

Perturbation Molecular Orbital (PMO) theory [63, 64] suggests that this interaction between n(X), $\sigma {\text{X}}^{\prime }-\text{H}$, and $\sigma^{*}{\text{X}}^{\prime }-\text{H}$ orbitals depends on their energies, the electronegativities of X and ${\text{X}}^{\prime }$, and the overlap of the orbitals. Morokuma's analysis of hydrogen bonds [65, 66] reveals how fragment electron densities change upon HB formation, forming the basis for modern EDA methods. These changes arise from either electrostatic deformation or orbital overlap, leading to electron sharing. It is clear that different chemical bond analysis methods consistently rely on at least a qualitative representation of atomic or fragment orbital overlap to assess the effectiveness of a chemical interaction.

In orbital interaction theory [63, 67], atomic orbital overlap is commonly used to assess the effectiveness of interactions between atoms or molecular fragments. Within this framework, the recently introduced chemical bond overlap model and its topological extension (OP/TOP) [68, 69] offer modern chemical bond descriptors. Initially applied to diatomic‐like systems [70] and Ln–L bonds in lanthanide complexes [71], the OP model has since proven versatile across diatomics [72, 73], molecular species [68, 69, 74, 75], coordination compounds [76, 77, 78, 79, 80, 81, 82, 83, 84], and solids [73, 85]. The OP model also effectively describes bonding in organic reaction systems, including equilibrium and transition states [74], yielding results consistent with QTAIM [12] and LVM [43, 86, 87] analyses.

Atomic orbital overlap reflects the interplay of electronic effects in molecular formation. As discussed by Levine and Head‐Gordon [88], and Martín and Francisco [89], it underlies the quantum mechanical nature of chemical bonds. The overlap density used in this work quantifies how atomic orbitals combine to form molecular orbitals and enables decomposition into one‐ and two‐center contributions, with the OP density representing the latter. Unlike models that infer electron distribution indirectly, the OP/TOP model provides complementary qualitative and quantitative descriptors sensitive to subtle changes in chemical environments.

This work presents a proof of concept for applying the OP/TOP model to hydrogen bonds, with comparisons to QTAIM, NBO, and LVM analyses. The chosen systems explore key factors affecting HB interactions. Systems **1**–**14**examine how O–H⋯O–R hydrogen bonds respond to increasing electron donation from R groups. Systems **15**–**18** vary the ${\text{R}}_{3}$C–H donor while keeping the $\text{N}{\left({\text{CH}}_{3}\right)}_{3}$ acceptor fixed. Systems **19**–**25** involve phenol complexes with ${\text{NH}}_{3}$ or $\text{N}{\left({\text{CH}}_{3}\right)}_{3}$, including systematic *para*‐substitutions. This diverse set enables a broad evaluation of the OP/TOP model's reliability.

## 2 Methodology

A detailed explanation of the OP/TOP descriptors is available in the literature [68, 69, 74, 79, 90], with a notable recent implementation presented in [91]. The following section offers a concise overview of the OP/TOP, QTAIM, and LVM descriptors, followed by a brief description of the computational methodology.

### 2.1 Overlap Properties

When typically orthogonal atomic orbitals combine into molecular orbitals, their overlap causes constructive or destructive interference, altering electron density across regions. Nodes form where overlapping orbitals cancel each other out. To bypass nodal and negative regions, the overlap density $\rho_{\text{OP}}(\vec{r})$ is calculated by numerically evaluating two‐center terms of each molecular orbital at various grid points $\vec{r}$ in space. This approach focuses exclusively on the positive (constructive) contributions, as detailed in our recent study [69]. The expression for $\rho_{\text{OP}}(\vec{r})$ is given by:

(1) $$\begin{array}{ll} \rho_{\text{OP}}(\vec{r}) & =2\overset{M}{\underset{l}{\sum }}n_{l}\overset{m}{\underset{i\in A}{\sum }}\overset{m}{\underset{j\in B}{\sum }}c_{li}c_{lj}\phi_{i}(\vec{r})\phi_{j}(\vec{r})\\ & \text{where}\;\vec{r}\in \{\vec{r}|\rho_{\text{OP}}(\vec{r})>0\}\; \end{array}$$

In this expression, l runs over all M molecular orbitals (spatial parts of the spin‐orbitals), with $n_{l}$ indicating the occupancy of each orbital. The parameter m represents the total number of atomic orbitals (AOs), $c_{i}$ are the coefficients from the Linear Combination of Atomic Orbitals (LCAO) expansion, and $\phi_{i}$ denotes the primitive or contracted functions that describe the AOs.

The overlap (OP) density, denoted as $\rho_{\text{OP}}(\vec{r})$, illustrates the two‐center (overlap) contribution to the total electron density in the molecule, reflecting how electrons are shared between atoms. The integrated overlap density, denoted as $\rho_{\text{OP}}$, is calculated by integrating Equation (1) over space [92], representing the electron density shared between the atoms involved in a bond.

The intra‐overlap Coulomb repulsion J, as described within the OP/TOP framework, is given by:

(2) $$J_{\text{OP}}^{\text{intra}}=\int \rho_{\text{OP}}(\vec{r_{1}})r_{12}^{-1}\rho_{\text{OP}}(\vec{r_{2}})dr_{1}dr_{2}$$

Here, $r_{12}$ represents the distance between points $r_{1}$ and $r_{2}$, where the overlap densities $\rho_{\text{OP}}(\vec{r_{1}})$ and $\rho_{\text{OP}}(\vec{r_{2}})$ are calculated. Generally, $\rho_{\text{OP}}$ tends to be larger in electron‐rich chemical bonds. Additionally, bonds with localized regions of concentrated OP density often exhibit higher values of $J_{\text{OP}}^{\text{intra}}$.

A deeper insight into the properties of $\rho_{\text{OP}}(\vec{r})$ can be achieved through a topological analysis of the overlap density, similar to the approach used in QTAIM. This method seeks to locate what are termed overlap critical points (OCPs) [69]. Recently, two TOP descriptors have been introduced: The density at the chemical bond OCP, denoted as $\rho_{\text{OCP}}$, and its Laplacian, $\nabla^{2}\rho_{\text{OCP}}$. OCPs located within chemical bonds are characterized by fully negative curvatures, which are determined by evaluating the Hessian of the density at these critical points. In general, a more tightly localized overlap density, $\rho_{\text{OP}}$, leads to more negative values of $\nabla^{2}\rho_{\text{OCP}}(\vec{r})$ and higher values of $\rho_{\text{OCP}}(\vec{r})$ [69].

### 2.2 QTAIM, NBO, and LVM Descriptors

For comparison, we employed key descriptors from three complementary theoretical frameworks.

From the Quantum Theory of Atoms in Molecules (QTAIM) [12], we used the electron density ($\rho_{\text{BCP}}$) and the Laplacian of the density ($\nabla^{2}\rho_{\text{BCP}}$) at bond critical points (BCPs), which provide insights into charge concentration or depletion in the bonding region. Additionally, the local energy density ($H_{\text{BCP}}$) is used to assess bond covalence or ionic character, based on the balance between stabilizing and destabilizing energy components at the BCP [15, 93].

From Natural Bond Orbital (NBO) analysis [94], we considered the second‐order perturbation stabilization energy ($\Delta E^{\left(2\right)}$) to quantify donor‐acceptor interactions between occupied and unoccupied NBOs. Furthermore, NBO occupations were examined to describe the electron distribution in chemically meaningful orbitals.

Lastly, the Local Vibrational Mode (LVM) theory [43, 86, 95, 96] provides local mode force constants ($k^{a}$), which are sensitive measures of bond strength. These constants are derived from normal mode analysis and allow for a localized interpretation of vibrational characteristics, making them effective descriptors of both strong and weak chemical interactions.

### 2.3 Computational Procedure

All geometry optimizations and subsequent calculations of normal vibrational frequencies were performed using Gaussian 09 [97] software. The geometries were fully optimized using Density Functional Theory (DFT) at the ωB97X‐D/def2‐TZVP [98, 99] level of theory. Each structure was confirmed as a true minimum through the analytical evaluation of harmonic frequencies. The electronic characteristics of O–H bonds, and O⋯H and N⋯H hydrogen bonds in various chemical environments were investigated using OP/TOP [68, 69, 91], QTAIM [12], NBO [37, 94, 99], and LVM [43, 86, 95, 96]. The descriptors were calculated using ChemBOS [90], Multiwfn [100], Gaussian 09 [97], and LModeA [101] software.

## 3 Results and Discussion

Table 1 provides a detailed summary of the chemical bond descriptors for all tested systems. The results are discussed in three sections: General trends and QTAIM descriptors, OP/TOP descriptors, and NBO descriptors. Finally, the synergy between OP/TOP, QTAIM, NBO, and LVM chemical bond analysis formalisms is examined.

**TABLE 1:** Results for systems **1**–**25** (see Figure 1): Bond distance r (in Å), overlap density $\rho_{\text{OP}}$ (in e), intra‐overlap repulsion $J_{\text{OP}}^{\text{intra}}$ (in eV), overlap critical point density $\rho_{\text{OCP}}$ (in $e/a_{0}^{3}$), Laplacian of $\rho_{\text{OCP}}$ at OCP $\nabla^{2}\rho_{\text{OCP}}$ (in $e/a_{0}^{5}$), local energy density ${\text{H}}_{\text{BCP}}$ (in eV/$a_{0}^{3}$), Laplacian $\nabla^{2}\rho_{\text{BCP}}$ (in $e/a_{0}^{5}$), electron density at BCP $\rho_{\text{BCP}}$ (in $e/a_{0}^{3}$), second perturbation energy $\Delta E^{\left(2\right)}$ for n(X)$\to \sigma^{*}{\text{X}}^{\prime }-\text{H}$ (in kcal/mol), and NBO occupation $occ^{\sigma^{*}(\text{X}\prime -\text{H})}$. O–H bonds in water molecule proton donors are labeled as D for the donating bond and P for the passive bond. Calculations were performed at the ωB97X‐D/def2‐TZVP level of theory (details in the Computational Procedure section).

#	Systems	Bond	r	$\rho_{\text{OP}}$	$J_{\text{OP}}^{\text{intra}}$	$\rho_{\text{OCP}}$	$\nabla^{2}\rho_{\text{OCP}}$	${\text{H}}_{\text{BCP}}$	$\nabla^{2}\rho_{\text{BCP}}$	$\rho_{\text{BCP}}$	$\Delta E^{\left(2\right)}$	$occ^{\sigma^{*}(\text{X}\prime -\text{H})}$
1	${\text{H}}_{2}\text{O}$	O–H	0.960	0.698	11.514	0.191	−1.676	−18.694	−2.463	0.363	—	0.0001
2	${\text{H}}_{2}\text{O}\text{⋯}{\text{H}}_{2}\text{O}$	O–H (P)	0.957	0.701	11.589	0.193	−1.724	−18.885	−2.476	0.367	—	—
		O–H (D)	0.966	0.701	11.463	0.191	−1.731	−18.422	−2.432	0.354	—	0.0151
		H⋯O	1.939	$3.76\cdot 10^{-2}$	$2.24\cdot 10^{-2}$	$0.33\cdot 10^{-2}$	$-3.43\cdot 10^{-2}$	$3.39\cdot 10^{-2}$	$8.90\cdot 10^{-2}$	$2.50\cdot 10^{-2}$	9.04	—
3	${\text{CH}}_{3}\text{HO}\text{⋯}{\text{H}}_{2}\text{O}$	O–H (P)	0.957	0.697	11.478	0.193	−1.733	−18.885	−2.476	0.367	—	—
		O–H (D)	0.968	0.696	11.326	0.190	−1.717	−18.340	−2.419	0.352	—	0.0181
		H⋯O	1.906	$6.36\cdot 10^{-2}$	$6.12\cdot 10^{-2}$	$0.53\cdot 10^{-2}$	$-5.50\cdot 10^{-2}$	$1.58\cdot 10^{-2}$	$9.50\cdot 10^{-2}$	$2.70\cdot 10^{-2}$	10.20	—
4	${\left({\text{CH}}_{3}\right)}_{2}\text{O}\text{⋯}{\text{H}}_{2}\text{O}$	O–H (P)	0.957	0.688	11.220	0.193	−1.744	−18.885	−2.477	0.367	—	—
		O–H (D)	0.968	0.677	10.881	0.189	−1.731	−18.313	−2.412	0.352	—	0.0199
		H⋯O	1.890	$8.75\cdot 10^{-2}$	$11.7\cdot 10^{-2}$	$0.71\cdot 10^{-2}$	$-7.05\cdot 10^{-2}$	$0.37\cdot 10^{-2}$	$9.80\cdot 10^{-2}$	$2.90\cdot 10^{-2}$	9.75	—
5	${\text{H}}_{2}\text{CO}\text{⋯}{\text{H}}_{2}\text{O}$	O–H (P)	0.957	0.691	11.267	0.192	−1.725	−18.939	−2.487	0.367	—	—
		O–H (D)	0.966	0.683	11.021	0.189	−1.703	−18.477	−2.439	0.355	—	0.0134
		H⋯O	2.001	$7.04\cdot 10^{-2}$	$7.28\cdot 10^{-2}$	$0.56\cdot 10^{-2}$	$-5.74\cdot 10^{-2}$	$5.88\cdot 10^{-2}$	$8.50\cdot 10^{-2}$	$2.20\cdot 10^{-2}$	6.15	—
6	${\text{CH}}_{3}\text{HCO}\text{⋯}{\text{H}}_{2}\text{O}$	O–H (P)	0.957	0.690	11.278	0.193	−1.735	−18.912	−2.482	0.367	—	—
		O–H (D)	0.967	0.684	11.000	0.188	−1.694	−18.368	−2.423	0.353	—	0.0164
		H⋯O	1.953	$7.63\cdot 10^{-2}$	$8.57\cdot 10^{-2}$	$0.60\cdot 10^{-2}$	$-6.05\cdot 10^{-2}$	$4.04\cdot 10^{-2}$	$9.10\cdot 10^{-2}$	$2.50\cdot 10^{-2}$	7.82	—
7	${\left({\text{CH}}_{3}\right)}_{2}\text{CO}\text{⋯}{\text{H}}_{2}\text{O}$	O–H (P)	0.957	0.690	11.315	0.193	−1.736	−18.885	−2.479	0.367	—	—
		O–H (D)	0.969	0.683	10.925	0.187	−1.665	−18.259	−2.413	0.351	—	0.0201
		H⋯O	1.914	$7.58\cdot 10^{-2}$	$8.66\cdot 10^{-2}$	$0.62\cdot 10^{-2}$	$-6.26\cdot 10^{-2}$	$2.38\cdot 10^{-2}$	$9.30\cdot 10^{-2}$	$2.70\cdot 10^{-2}$	9.98	—
8	${\text{NH}}_{3}\text{⋯}{\text{H}}_{2}\text{O}$	O–H (P)	0.957	0.702	11.588	0.194	−1.742	−18.857	−2.466	0.368	—	—
		O–H (D)	0.972	0.711	11.745	0.190	−1.716	−17.987	−2.368	0.347	—	0.0264
		H⋯N	1.971	$1.13\cdot 10^{-2}$	$0.26\cdot 10^{-2}$	$0.250\cdot 10^{-2}$	$-1.82\cdot 10^{-2}$	$-1.80\cdot 10^{-2}$	$7.60\cdot 10^{-2}$	$2.80\cdot 10^{-2}$	14.73	—
9	$({\text{CH}}_{3}){\text{NH}}_{2}\text{⋯}{\text{H}}_{2}\text{O}$	O–H (P)	0.957	0.694	11.464	0.193	−1.751	−18.857	−2.465	0.368	—	—
		O–H (D)	0.975	0.695	11.181	0.188	−1.704	−17.769	−2.335	0.343	—	0.0317
		H⋯N	1.929	$5.77\cdot 10^{-2}$	$4.96\cdot 10^{-2}$	$0.46\cdot 10^{-2}$	$-2.40\cdot 10^{-2}$	$-5.27\cdot 10^{-2}$	$8.00\cdot 10^{-2}$	$3.20\cdot 10^{-2}$	15.75	—
10	${\left({\text{CH}}_{3}\right)}_{2}$NH$\text{⋯}{\text{H}}_{2}$O	O–H (P)	0.957	0.685	11.164	0.193	−1.766	−18.857	−2.467	0.368	—	—
		O–H (D)	0.976	0.672	10.604	0.185	−1.688	−17.660	−2.316	0.342	—	0.0335
		H⋯N	1.906	$10.6\cdot 10^{-2}$	$16.2\cdot 10^{-2}$	$0.68\cdot 10^{-2}$	$-2.85\cdot 10^{-2}$	$-7.37\cdot 10^{-2}$	$8.30\cdot 10^{-2}$	$3.40\cdot 10^{-2}$	15.36	—
11	${\left({\text{CH}}_{3}\right)}_{3}\text{N}\text{⋯}{\text{H}}_{2}\text{O}$	O–H (P)	0.957	0.692	11.349	0.194	−1.769	−18.830	−2.464	0.368	—	—
		O–H (D)	0.977	0.655	10.206	0.183	−1.648	−17.606	−2.306	0.341	—	0.0331
		H⋯N	1.900	$16.1\cdot 10^{-2}$	$36.6\cdot 10^{-2}$	$0.91\cdot 10^{-2}$	$-3.10\cdot 10^{-2}$	$-8.89\cdot 10^{-2}$	$8.20\cdot 10^{-2}$	$3.50\cdot 10^{-2}$	14.59	—
12	$\text{HCN}\text{⋯}{\text{H}}_{2}\text{O}$	O–H (P)	0.957	0.707	11.788	0.194	−1.740	−18.885	−2.477	0.367	—	—
		O–H (D)	0.963	0.713	11.806	0.191	−1.675	−18.721	−2.475	0.359	—	0.0067
		H⋯N	2.138	$3.40\cdot 10^{-2}$	$1.58\cdot 10^{-2}$	$0.17\cdot 10^{-2}$	$-0.69\cdot 10^{-2}$	$6.93\cdot 10^{-2}$	$6.00\cdot 10^{-2}$	$1.60\cdot 10^{-2}$	3.68	—
13	${\text{CH}}_{3}\text{CN}\text{⋯}{\text{H}}_{2}\text{O}$	O–H (P)	0.957	0.710	11.801	0.195	−1.748	−18.885	−2.473	0.368	—	—
		O–H (D)	0.964	0.716	11.810	0.189	−1.639	−18.613	−2.463	0.357	—	0.0095
		H⋯N	2.082	$4.29\cdot 10^{-2}$	$2.54\cdot 10^{-2}$	$0.23\cdot 10^{-2}$	$-0.91\cdot 10^{-2}$	$6.39\cdot 10^{-2}$	$6.70\cdot 10^{-2}$	$1.90\cdot 10^{-2}$	4.95	—
14	${\text{CH}}_{3}{\text{CH}}_{2}\text{CN}\text{⋯}{\text{H}}_{2}\text{O}$	O–H (P)	0.957	0.711	11.869	0.195	−1.750	−18.857	−2.473	0.368	—	—
		O–H (D)	0.964	0.717	11.798	0.188	−1.629	−18.613	−2.462	0.357	—	0.0096
		H⋯N	2.079	$4.41\cdot 10^{-2}$	$2.67\cdot 10^{-2}$	$0.23\cdot 10^{-2}$	$-0.92\cdot 10^{-2}$	$6.31\cdot 10^{-2}$	$6.70\cdot 10^{-2}$	$1.90\cdot 10^{-2}$	5.04	—
15	${\text{HCF}}_{3}$	C–H	1.091	0.870	15.359	0.159	−1.137	−2.161	−1.193	0.306	—	0.0588
16	${\left({\text{CH}}_{3}\right)}_{3}\text{N}\text{⋯}{\text{HCF}}_{3}$	C–F (P)	1.337	0.678	10.493	0.178	−1.701	−3.059	−0.394	0.286	—	—
		C–H (D)	1.095	0.840	14.676	0.158	−1.118	−2.183	−1.211	0.306	—	0.0640
		H⋯N	2.242	$9.81\cdot 10^{-2}$	$12.4\cdot 10^{-2}$	$0.53\cdot 10^{-2}$	$-3.59\cdot 10^{-2}$	$0.94\cdot 10^{-2}$	$5.33\cdot 10^{-2}$	$1.87\cdot 10^{-2}$	31.93	—
17	${\text{HCCl}}_{3}$	C–H	1.083	0.903	16.034	0.162	−1.455	−2.126	−1.141	0.301	—	0.04483
18	${\left({\text{CH}}_{3}\right)}_{3}\text{N}\text{⋯}{\text{HCCl}}_{3}$	C–Cl (P)	1.771	0.750	10.364	0.153	−1.214	−0.9193	−0.2759	0.1960	—	—
		C–H (D)	1.093	0.807	13.660	0.092	−0.428	−2.106	−1.139	0.297	—	0.0579
		H⋯N	2.072	0.167	$36.9\cdot 10^{-2}$	$0.98\cdot 10^{-2}$	$-6.59\cdot 10^{-2}$	$0.25\cdot 10^{-2}$	$6.966\cdot 10^{-2}$	$2.623\cdot 10^{-2}$	24.74	—
19	${\text{HOC}}_{6}{\text{H}}_{5}$	O–H	0.958	0.708	11.837	0.200	−1.819	−4.795	−2.567	0.368	—	0.0057
20	${\text{NH}}_{3}\text{⋯}{\text{HOC}}_{6}{\text{H}}_{5}$	O–C (P)	1.345	0.819	14.681	0.177	−1.285	−3.296	−0.608	0.307	—	—
		O–H (D)	0.980	0.698	11.334	0.189	−1.729	−4.353	−2.316	0.337	—	0.0483
		H⋯N	1.872	$1.69\cdot 10^{-2}$	$0.60\cdot 10^{-2}$	$0.42\cdot 10^{-2}$	$-2.97\cdot 10^{-2}$	$-2.48\cdot 10^{-2}$	$8.37\cdot 10^{-2}$	$3.60\cdot 10^{-2}$	27.32	—
21	${\left({\text{CH}}_{3}\right)}_{3}\text{N}\text{⋯}{\text{HOC}}_{6}{\text{H}}_{5}$	O–C (P)	1.345	0.818	14.467	0.177	−1.287	−3.296	−0.606	0.307	—	—
		O–H (D)	0.987	0.625	9.506	0.180	−1.638	−4.220	−2.226	0.330	—	0.0552
		H⋯N	1.810	$22.2\cdot 10^{-2}$	$70.4\cdot 10^{-2}$	$1.36\cdot 10^{-2}$	$-4.54\cdot 10^{-2}$	$-5.10\cdot 10^{-2}$	$8.60\cdot 10^{-2}$	$4.35\cdot 10^{-2}$	23.41	—
22	${\text{HOC}}_{6}{\text{H}}_{4}\text{N}{\left({\text{CH}}_{3}\right)}_{2}$	O–H	0.958	0.709	11.915	0.200	−1.827	−4.794	−2.563	0.369	—	0.0053
23	${\text{NH}}_{3}\text{⋯}{\text{HOC}}_{6}{\text{H}}_{4}\text{N}{\left({\text{CH}}_{3}\right)}_{2}$	O–C (P)	1.352	0.822	14.514	0.174	−1.251	−3.205	−0.612	0.302	—	—
		O–H (D)	0.978	0.703	11.478	0.190	−1.751	−4.393	−2.338	0.340	—	0.0444
		H⋯N	1.892	$1.36\cdot 10^{-2}$	$0.39\cdot 10^{-2}$	$0.36\cdot 10^{-2}$	$-2.68\cdot 10^{-2}$	$-1.99\cdot 10^{-2}$	$8.24\cdot 10^{-2}$	$3.43\cdot 10^{-2}$	22.87	—
24	${\text{HOC}}_{6}{\text{H}}_{4}{\text{NO}}_{2}$	O–H	0.959	0.702	11.712	0.199	−1.802	−4.792	−2.572	0.367	—	0.0058
25	${\text{NH}}_{3}\text{⋯}{\text{HOC}}_{6}{\text{H}}_{4}{\text{NO}}_{2}$	O–C (P)	1.333	0.871	16.078	0.184	−1.340	−3.448	−0.599	0.316	—	—
		O–H (D)	0.986	0.682	10.864	0.184	−1.672	−4.248	−2.256	0.330	—	0.0579
		H⋯N	1.824	$3.04\cdot 10^{-2}$	$1.92\cdot 10^{-2}$	$0.60\cdot 10^{-2}$	$-3.78\cdot 10^{-2}$	$-3.90\cdot 10^{-2}$	$8.62\cdot 10^{-2}$	$4.03\cdot 10^{-2}$	30.27	—

### 3.1 Expected Trends and QTAIM Descriptors

Freindorf and colleagues [64] previously reported QTAIM and local stretching mode analyses for a broad range of HB interactions, including systems **2**–**6**. The QTAIM results obtained in the present work are consistent with those reported by Freindorf et al. Both studies show similar trends. However, in this work, the ${\text{H}}_{\text{BCP}}$ values are positive and close to zero for most hydrogen bonds. In contrast, Freindorf reported negative, near‐zero values for systems **2**–**6**. This discrepancy is likely attributable to the slight difference in basis sets used, with Freindorf employing aug‐cc‐pVTZ and the present work utilizing def2‐TZVP. In a recent study [69], the dependence of QTAIM descriptors on the choice of basis sets for covalent bonds was analyzed, revealing that basis sets with additional polarization functions can cause an accumulation of electron density in the bonding region. Negative values of ${\text{H}}_{\text{BCP}}$ are frequently attributed to this increased charge density at the bond critical point [18, 102, 103, 104].

**FIGURE 1:**
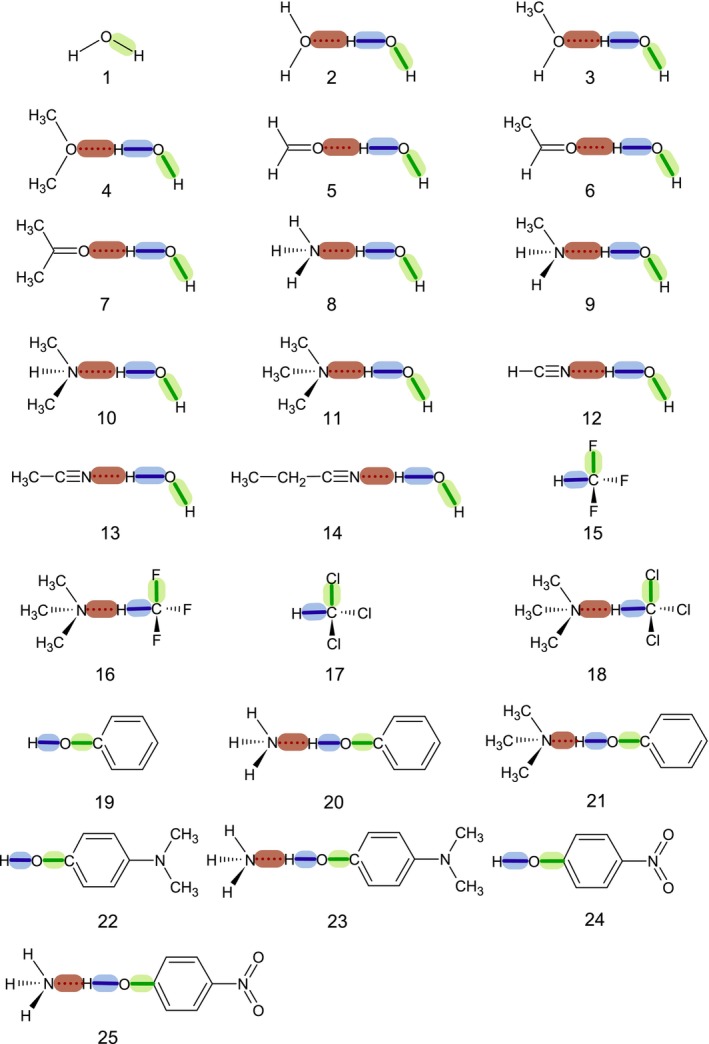
Schematic representation of the studied molecular systems. Hydrogen bonds, O–H or C–H proton donors, and O–H, C–F, C–Cl, or C–O passive bonds are highlighted in red, blue, and green, respectively.

In systems **2**–**4**, the gradual addition of $-{\text{CH}}_{3}$ electron‐donating groups does not significantly affect the QTAIM descriptors for the O–H(P) bonds. This observation also holds for systems **8**–**11** and **12**–**14**, where only minimal variations are detected. However, the QTAIM descriptors for O–H(D) bonds and HBs in systems **2**–**14** exhibit greater sensitivity, showing a clear trend. As anticipated, the inclusion of electron‐donating groups (R) bonded to the X atom in the ${\text{X}}^{\prime }\text{‐H}\text{⋯}\text{X}$–R interaction enhances the electron density at n(X), promoting its interaction with $\sigma^{*}{\text{X}}^{\prime }\text{‐H}$. Adding $-{\text{CH}}_{3}$ groups decreases ${\text{H}}_{\text{BCP}}$ and increases $\rho_{\text{BCP}}$ for the hydrogen bonds. This indicates greater electron density at the bond critical point.

**FIGURE 2:**
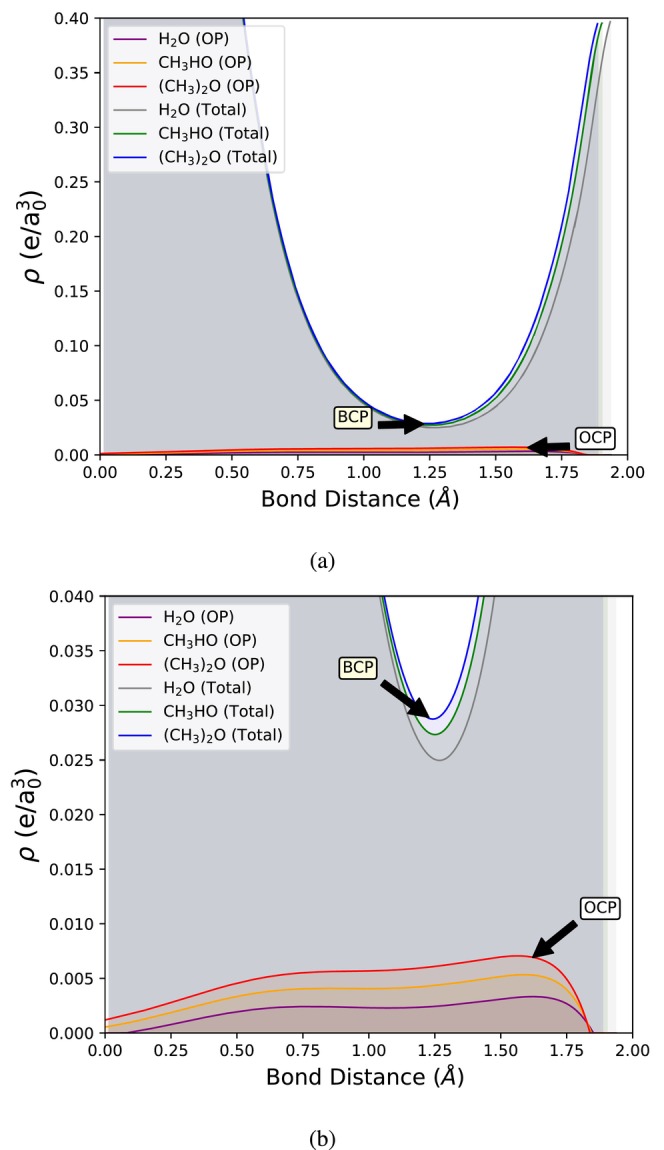
Profiles of the total electron density $\rho (\vec{r})$ and the overlap density $\rho_{\text{OP}}(\vec{r})$ along the hydrogen bond axis X⋯H in (a) 0.00–0.40 $e/a_{0}^{3}$, and (b) 0.00–0.04 $e/a_{0}^{3}$ ranges. The HB acceptor atom X is positioned at 0.0 Å, while the HB donor atom is located near 1.9 Å, with slight variation.

The Laplacian $\nabla^{2}\rho_{\text{BCP}}$ increases with the addition of $-{\text{CH}}_{3}$ groups across all groups of test systems, suggesting a concentration of electron density at the attractor linked to the hydrogen bond BCP. This is likely due to the rising electron density at the H–${\text{X}}^{\prime }$ fragment, which increases the curvature of the total electron density at the BCP, ultimately leading to a higher $\nabla^{2}\rho_{\text{BCP}}$. As a result, this behavior might falsely indicate a depletion of charge at the hydrogen bond, even though the electron density is, in fact, increasing. Figure 2a illustrates this increasing electron density at the H–${\text{X}}^{\prime }$ fragment as more $-{\text{CH}}_{3}$ electron‐donating groups are introduced connected to the X atom.

Systems **15**–**18** involve unconventional ${\text{R}}_{3}\text{C}-\text{H}\text{⋯}\text{N}{\left({\text{CH}}_{3}\right)}_{3}$ hydrogen bonds, where the proton donor bond environment varies from $\text{H}-{\text{CF}}_{3}$ to $\text{H}-{\text{CCl}}_{3}$. It is well known that ${\text{HCCl}}_{3}$ (pKa=15.7) [105] is a stronger acid compared to ${\text{HCF}}_{3}$ (pKa=25–28) [106]. This reversed trend relative to electronegativity is generally attributed to the greater stability of the carbanion formed in ${\text{CHCl}}_{3}$ [107]. From the perspective of bond polarity and stability, fluorine atoms make the carbon center highly electron‐deficient, thereby increasing the polarity of the H–C bond in ${\text{CHF}}_{3}$. Consequently, the measured H–C bond dissociation energy in ${\text{CHF}}_{3}$ (106.7 kcal/mol) is higher than that in ${\text{CHCl}}_{3}$ (95.8 kcal/mol) [108].

Notably, as the system changes from isolated $\text{H}-{\text{CF}}_{3}$ (system **15**) to $\text{H}-{\text{CCl}}_{3}$ (system **17**), both ${\text{H}}_{\text{BCP}}$ and $\nabla^{2}\rho_{\text{BCP}}$ become less negative for the H–C bond. This behavior contradicts expectations, as the more electronegative F atoms are expected to further polarize the H–C bond compared to Cl atoms. When the hydrogen bond ${\left({\text{CH}}_{3}\right)}_{3}\text{N}\text{⋯}{\text{HCF}}_{3}$ (system **16**) is formed, ${\text{H}}_{\text{BCP}}$ and $\nabla^{2}\rho_{\text{BCP}}$ for the proton donor H–C bond become more negative than the isolated $\text{H}-{\text{CF}}_{3}$. This indicates an increase in covalence and charge concentration in the bond region. Conversely, the formation of the hydrogen bond ${\left({\text{CH}}_{3}\right)}_{3}\text{N}\text{⋯}{\text{HCCl}}_{3}$ (system **18**) leads to the expected less negative values of ${\text{H}}_{\text{BCP}}$ and $\nabla^{2}\rho_{\text{BCP}}$. From the perspective of the N⋯H–C hydrogen bonds, QTAIM indicates that, compared to ${\left({\text{CH}}_{3}\right)}_{3}\text{N}\text{⋯}{\text{HCF}}_{3}$, the ${\left({\text{CH}}_{3}\right)}_{3}\text{N}\text{⋯}{\text{HCCl}}_{3}$ interaction is less ionic (as evidenced by a less positive ${\text{H}}_{\text{BCP}}$) and exhibits more pronounced charge depletion in the hydrogen bond region (as indicated by a more positive $\nabla^{2}\rho_{\text{BCP}}$).

Systems **19**–**25** include substituted phenols that act as proton donors. Their QTAIM descriptors follow trends similar to those observed in systems **2**–**14**. The formation of the hydrogen bond between ${\text{NH}}_{3}$ and ${\text{HOC}}_{6}{\text{H}}_{5}$ (systems **19** and **20**) causes the values of ${\text{H}}_{\text{BCP}}$ and $\nabla^{2}\rho_{\text{BCP}}$ for the O–H bond to become less negative Additionally, comparing systems **20** and **21**, the inclusion of electron‐donating $-{\text{CH}}_{3}$ groups attached to nitrogen accentuates this effect.

Systems **22**–**25** provide a detailed perspective on how variations in the chemical environment influence the proton‐donor bond. The electron‐donating $-\text{N}{\left({\text{CH}}_{3}\right)}_{2}$ group at the *para* position increases electron density in the aromatic ring. This reduces the π‐character of the O–C (P) bond, weakens the O–H group's ability to donate charge to the ring, and makes it a less effective proton donor in hydrogen bonds. A comparison of systems **20**, **23**, and **25** shows that QTAIM descriptors reflect these effects. From system **20** to system **23**, the covalent character of the O–C (P) bond decreases (indicated by a less negative ${\text{H}}_{\text{BCP}}$). At the same time, charge accumulates in the bond region (indicated by a more negative $\nabla^{2}\rho_{\text{BCP}}$). The opposite trend is observed in system **25**, where the electron‐withdrawing $-{\text{NO}}_{2}$ substituent enhances the π‐character of the O–C (P) bond. QTAIM descriptors also indicate that O–H (D) bonds are more covalent and exhibit greater charge concentration when the electron‐donating $-\text{N}{\left({\text{CH}}_{3}\right)}_{2}$ group is present, whereas the opposite effect is observed for the electron‐withdrawing $-{\text{NO}}_{2}$ substituent. Finally, in the ${\text{NH}}_{3}\text{⋯}{\text{HOC}}_{6}{\text{H}}_{4}\text{N}-\text{R}$ hydrogen bond interaction, QTAIM descriptors suggest a decrease in covalent character (as evidenced by a less negative ${\text{H}}_{\text{BCP}}$) when R = $-\text{N}{\left({\text{CH}}_{3}\right)}_{2}$ (system **23**) and an increase when R = $-{\text{NO}}_{2}$ (system **25**), both relative to R = H (system **20**).

### 3.2 NBO Analysis

NBO analysis reveals that, in general, the inclusion of $-{\text{CH}}_{3}$ groups attached to X atom increases the second‐order perturbation energies $\Delta E^{\left(2\right)}$ for the n(X)$\to \sigma^{*}{\text{X}}^{\prime }\text{‐H}$ NBOs interaction in systems **2**‐**14**. Exceptions are observed for systems **4**, **10**, and **11**, likely due to steric interactions unrelated to the hydrogen bond. Similarly, the occupation of the $\sigma^{*}{\text{X}}^{\prime }\text{‐H}$ orbital tends to increase with the inclusion of $-{\text{CH}}_{3}$ groups attached to X, indicating a weakening of the ${\text{X}}^{\prime }\text{‐H}$ bond upon hydrogen bond formation.

For systems **15**–**18**, the $\sigma^{*}(\text{C}-\text{H})$ occupation increases upon hydrogen bond formation from **15** to **16**, and from **17** to **18**. When the hydrogen bond ${\left({\text{CH}}_{3}\right)}_{3}\text{N}\text{⋯}{\text{HCF}}_{3}$ (system **16**) is formed, an increase of 0.0052 (compared to isolated ${\text{HCF}}_{3}$) in $\sigma^{*}(\text{C}-\text{H})$ occupation is observed, indicating a weakening of the C–H bond. The same is observed for ${\left({\text{CH}}_{3}\right)}_{3}\text{N}\text{⋯}{\text{HCCl}}_{3}$ (system **18**), with an increase of 0.013 (compared to isolated ${\text{HCCl}}_{3}$) in $\sigma^{*}(\text{C}-\text{H})$ occupation. When systems **16** and **18** are compared, a decrease in the $\sigma^{*}(\text{C}-\text{H})$ occupation is observed, along with a decreased $\Delta E^{\left(2\right)}$ energy, indicating a more stable and stronger hydrogen bond for ${\left({\text{CH}}_{3}\right)}_{3}\text{N}\text{⋯}{\text{HCF}}_{3}$ interaction. This trend is not consistent with the pKa and bond dissociation energies from the literature discussed above, which suggest a more effective ${\left({\text{CH}}_{3}\right)}_{3}\text{N}\text{⋯}{\text{HCCl}}_{3}$ interaction.

Systems **19**–**25** exhibit a consistent trend in $\sigma^{*}(\text{C}-\text{H})$ occupations. In general, hydrogen bond formation increases $\sigma^{*}$(O–H) occupation, indicating a more effective n(X)$\to \sigma^{*}{\text{X}}^{\prime }\text{‐H}$ interaction. From system **19** to **20**, the addition of $-{\text{CH}}_{3}$ groups attached to X atom confers additional $\sigma^{*}$(O–H) occupation, aligning with observations from systems **2**–**14**. Introducing the electron‐donating $-\text{N}{\left({\text{CH}}_{3}\right)}_{2}$ group at the *para* position (comparing systems **20** and **23**) decreases $\sigma^{*}$(O–H) occupation. This aligns with the predicted reduced proton donor effectiveness of O–H (D) in hydrogen bonds. Conversely, the enhancement of O–H (D) as a proton donor by the electron‐withdrawing $-{\text{NO}}_{2}$ substituent (system **25**) is reflected in the increased $\sigma^{*}$(O–H) NBO occupation.

### 3.3 OP/TOP Perspective

**FIGURE 3:**
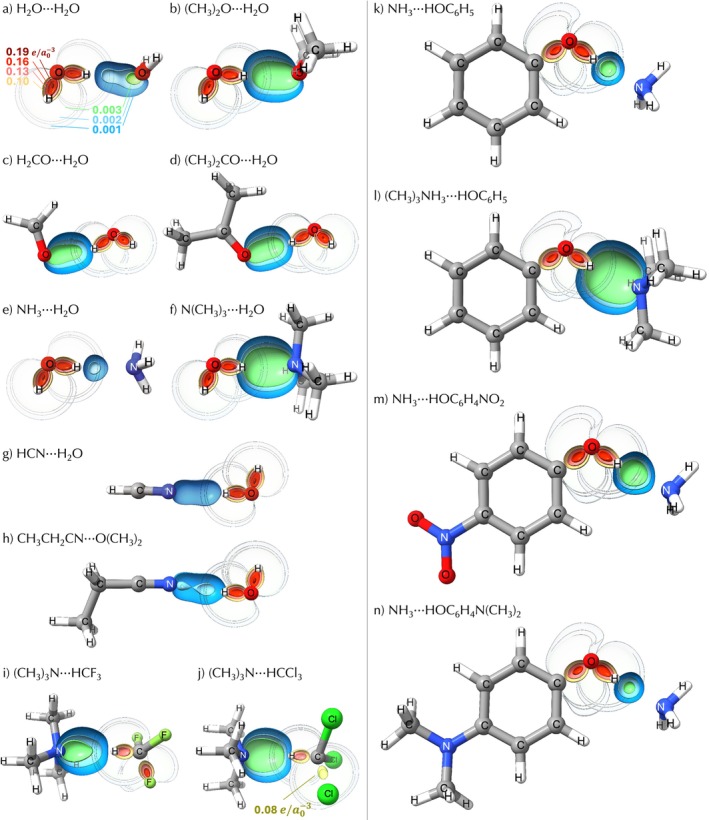
OP density maps $\rho_{\text{OP}}(\vec{r})$ for selected systems, displaying values ranging from 0.001 to 0.19 $e/a_{0}^{3}$, using a red‐green‐blue color scheme as illustrated in (a) and (j). Isovalues between 0.001 and 0.003 $e/a_{0}^{3}$ for O–H bonds are shown with higher transparency for improved visualization. Calculations were conducted at the ωB97X‐D/def2‐TZVP level of theory. The maps were generated with ChimeraX software [109, 110, 111, 112], utilizing a clipping plane along the O–H and hydrogen bond planes.

The OP/TOP model reveals how orbital interactions underlying hydrogen bonding evolve as the chemical environment is systematically varied across systems **1**–**25**. This is demonstrated in Table 1 and Figure 3.

This work aims to demonstrate that the OP/TOP model has potential utility in hydrogen bond interactions, where the orbital interactions are relatively weak, leading to reduced fragment orbital overlap. In such cases, the OP/TOP descriptors offer a clearer visualization of how these groups enhance orbital overlap, providing a more straightforward interpretation of the strengthening hydrogen bond interaction compared to traditional electron density descriptors.

Figure 2b shows the electron density profiles (total and overlap) along the hydrogen bond axis, for systems **2**–**4**, highlighting the BCPs and OCPs. It is evident that the gradual addition of $-{\text{CH}}_{3}$ electron‐donating groups increases the extent of orbital interaction, which is reflected in a more pronounced overlap density. More negative values of $\nabla^{2}\rho_{\text{OCP}}$ and higher values of $\rho_{\text{OCP}}$ indicate more concentrated overlap densities as $-{\text{CH}}_{3}$ groups are gradually added to the X atom. Moreover, $\rho_{\text{OP}}(\vec{r})$ appears slightly less spread as it becomes more concentrated toward the H atom.

The OP/TOP descriptors reflect the enhancement of electron density at n(X) due to the introduction of $-{\text{CH}}_{3}$ electron‐donating groups attached to the X atom. The increase in OP density is consistently observed across all groups of test systems (**2**–**4**, **5**–**7**, **8**–**11**, and **12**–**14**) as illustrated in Table 1 and Figure 3a–h. Additionally, the OP/TOP descriptors for the adjacent O–H(D) and O–H(P) bonds capture the electron density rearrangement triggered by the n(X)$\to \sigma^{*}{\text{X}}^{\prime }\text{‐H}$ orbital interaction. With the inclusion of $-{\text{CH}}_{3}$ electron‐donating groups in systems **2**–**14**, the X⋯H hydrogen bond becomes more electron‐rich, enhancing the n(X)$\to \sigma^{*}{\text{X}}^{\prime }\text{‐H}$ electron donation. This, in turn, is expected to weaken the O–H(D) bond, leading to a reduction in electron sharing, as indicated by the lower OP density ($\rho_{\text{OP}}$) in the O–H(D) bond. Conversely, the electron sharing increases (higher $\rho_{\text{OP}}$) in the O–H(P) bond, as demonstrated in Table 1 and Figure 3.

Figure 3 presents the OP density maps for the X⋯H, O–H(D), and O–H(P) bonds. It is evident that, as we transition from ${\text{H}}_{2}\text{O}\text{⋯}{\text{H}}_{2}\text{O}$ to ${\left({\text{CH}}_{3}\right)}_{2}\text{O}\text{⋯}{\text{H}}_{2}\text{O}$ (Figure 3a,b), the overlap density $\rho_{\text{OP}}(\vec{r})$ extension increases for the same isovalue. A similar trend is observed when comparing ${\text{NH}}_{3}\text{⋯}{\text{H}}_{2}$O to ${\left({\text{CH}}_{3}\right)}_{3}\text{N}\text{⋯}{\text{H}}_{2}$O (Figure 3e,f). In line with these observations, the OP density for O–H(D) bonds decreases, while it increases for O–H(P) bonds, which is consistent with the $\rho_{\text{OP}}$ values reported in Table 1.

OP/TOP descriptors for systems **15**–**18** indicate a decrease in overlap density for C–H bonds upon hydrogen bond formation in both $\text{H}-{\text{CF}}_{3}$ and $\text{H}-{\text{CCl}}_{3}$. Comparing ${\left({\text{CH}}_{3}\right)}_{3}\text{N}\text{⋯}{\text{HCF}}_{3}$ (system **16**) with ${\left({\text{CH}}_{3}\right)}_{3}\text{N}\text{⋯}{\text{HCCl}}_{3}$ (system **18**), the OP/TOP model captures the expected stronger N⋯H–C hydrogen bond interaction when Cl atoms are attached to C, as evidenced by the significantly higher $\rho_{\text{OP}}$. This trend is clearly illustrated in the OP density maps in Figure 3i,j. The greater propensity of $\text{H}-{\text{CCl}}_{3}$ to act as a proton donor is reflected in a reduction of electron sharing in the C–H (D) bond, as indicated by the lower OP density ($\rho_{\text{OP}}$) for ${\left({\text{CH}}_{3}\right)}_{3}\text{N}\text{⋯}{\text{HCCl}}_{3}$ (system **18**) compared to ${\left({\text{CH}}_{3}\right)}_{3}\text{N}\text{⋯}{\text{HCF}}_{3}$ (system **16**).

Systems **20** and **21** follow a trend similar to systems **2**–**14**, where the inclusion of $-{\text{CH}}_{3}$ electron‐donating groups attached to the N atom increases OP density. The OP/TOP model captures how the electron‐donating substituent $-\text{N}{\left({\text{CH}}_{3}\right)}_{2}$ saturates the ring, reducing the effectiveness of substituted phenol as a proton donor in hydrogen bonds. Comparing systems **20** and **23**, the introduction of the $-\text{N}{\left({\text{CH}}_{3}\right)}_{2}$ group in the para position leads to an increase in O–H (D) $\rho_{\text{OP}}$, consistent with its weakened proton‐donating ability. Conversely, the electron‐withdrawing $-{\text{NO}}_{2}$ substituent in system **25** enhances the O–H (D) proton donor ability, reflected in a decrease in its OP density. These effects are directly mirrored in the hydrogen bonds, where the electron‐withdrawing substituent increases O–H⋯N OP density, opposite to the decrease observed with the electron‐donating substituent, as shown in Figure 3k,m,n.

It is important to emphasize that the OP/TOP approach, which decomposes the total one‐particle electron density into overlap (two‐center) and atomic (one‐center) contributions, provides a deconvolution of the electron density. The OP/TOP model specifically aims to extract chemical insights from the overlap density portion. As a result, descriptors based solely on the total electron density topology, such as $\nabla^{2}\rho_{\text{BCP}}$, can lead to misleading interpretations, particularly in cases with weak orbital interactions, such as in hydrogen bond formation. Notably, the trend of increasing $\nabla^{2}\rho_{\text{BCP}}$ with the inclusion of electron‐donating groups remains consistent across different basis sets (see Tables S1–S3 in the supporting information). It is also important to acknowledge that OP/TOP descriptors inherently exhibit basis‐set dependence due to their Mulliken‐like partitioning (see Tables S4–S7). However, despite this, the OP/TOP descriptors maintain the same trend when the same basis set is used, thereby validating the trends discussed herein.

### 3.4 Synergy Between OP/TOP, QTAIM, NBO, and LVM

**FIGURE 4:**
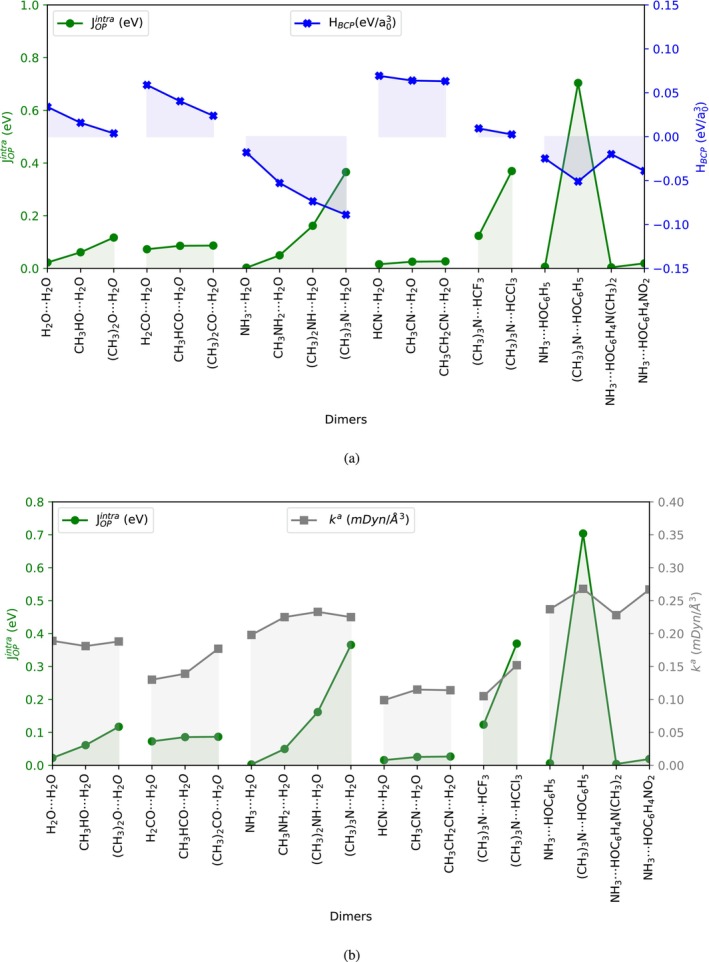
Plots of (a) $J_{\text{OP}}^{\text{intra}}$ (left axis) and ${\text{H}}_{\text{BCP}}$ (right axis), and (b) $J_{\text{OP}}^{\text{intra}}$ (left axis) and ${\text{k}}^{a}$ (right axis) for X⋯H hydrogen bonds across the studied systems. Calculations were performed using the ωB97X‐D/def2‐TZVP level of theory.

When compared with QTAIM, the OP/TOP descriptors exhibit consistent trends. In general, bonds expected to have decreased electron sharing show less negative ${\text{H}}_{\text{BCP}}$, indicating reduced covalence. From the OP/TOP perspective, this situation is captured by smaller $\rho_{\text{OP}}$ values, as observed across the groups of systems **2**–**4**, **5**–**7**, **8**–**11**, **12**–**14**, **15**–**18**, and **19**–**25**. As illustrated in Figure 4a, $J_{\text{OP}}^{\text{intra}}$ increases within each group of test systems, while ${\text{H}}_{\text{BCP}}$ decreases. The reduction in ${\text{H}}_{\text{BCP}}$ indicates an increase in the covalent character of the hydrogen bond, aligning with the expected behavior when $-{\text{CH}}_{3}$ groups are introduced. These electron‐donating groups enhance n(X) donation to the hydrogen bond. The rise in $J_{\text{OP}}^{\text{intra}}$, which reflects the integrated potential energy of the overlap density, likely explains the observed decrease in ${\text{H}}_{\text{BCP}}$, as it negatively accounts for the potential energy at the BCP. This relationship reinforces $J_{\text{OP}}^{\text{intra}}$ as a valuable indicator of bond strength and interaction.

Interestingly, OP/TOP descriptors for hydrogen bond interactions align well with the concept that $\sigma^{*}$(X–H) occupation increases upon hydrogen bond formation. As shown in Table 1, within each group of studied systems, an increase in $\sigma^{*}$(X–H) occupation indicates a weakened X–H bond, resulting from a more effective hydrogen bond interaction of the type n(X)$\to \sigma^{*}{\text{X}}^{\prime }\text{‐H}$. Furthermore, according to Alabugin [60], an increase in the population of the $\sigma^{*}$(X–H) antibonding orbital contributes to a direct overlap with the n(X) lone‐pair orbital. This trend is precisely captured by OP/TOP $\rho_{\text{OP}}$ integrated values and maps, even though OP density is derived from canonical (rather than natural) orbitals and considers contributions from all occupied canonical orbitals.

According to the Localized Vibrational Mode (LVM) theory, local mode force constants ($k^{a}$) are sensitive to electronic structure variations induced by substituents [43]. Independent of atomic masses, these constants capture purely electronic effects. The local stretching force constant $k_{n}^{a}$ for a bond A–B in an R–A–B–R' system measures the intrinsic bond strength, linked to the second derivative of the molecular energy concerning the internal coordinate $q_{n}$. Zou and Cremer [113] showed that by approximating the Born‐Oppenheimer potential energy surface with a Morse potential, while freezing the electron density during dissociation, bond strength becomes proportional to $k^{a}$. Thus, $k^{a}$ is a universal indicator of a bond's intrinsic strength, as observed through vibrational spectroscopy.

OP/TOP descriptors have previously been compared with LVM‐derived bond strength for conventional chemical bonds using MCSCF [114] and DFT [74, 92] calculations. In the specific case of hydrogen bonds examined here, as illustrated in Figure 4b, both $J_{\text{OP}}^{\text{intra}}$ (resulting from an increase in $\rho_{\text{OP}}$) and $k^{a}$ generally increase across each group of test systems. This correlation suggests that stronger hydrogen bonds, as indicated by LVM, are associated with greater shared electron density.

General correlations between values reported in Table 1 are shown in the heat maps of Figures S1–S3, highlighting distinct patterns for passive, donor, and hydrogen bonds. For passive and donor bonds (Figures S1 and S2), most descriptors are strongly correlated, particularly QTAIM ($\rho_{\text{BCP}}$, $\nabla^{2}\rho_{\text{BCP}}$, ${\text{H}}_{\text{BCP}}$) and OP/TOP descriptors ($\rho_{\text{OP}}$, $\rho_{\text{OCP}}$, $J_{\text{OP}}^{\text{intra}}$) with correlation coefficients often exceeding 0.7 or 0.8. The NBO occupation ${\text{occ}}_{\sigma^{*}}$ correlates well with $\nabla^{2}\rho_{\text{BCP}}$ and $H_{\text{BCP}}$, supporting its role in donor‐acceptor interactions. In contrast, hydrogen bonds (Figure S3) show weaker and more scattered correlations. Notably, $\rho_{\text{BCP}}$, ${\text{H}}_{\text{BCP}}$, and $\Delta E^{\left(2\right)}$ are negatively correlated with distance, indicating sensitivity to bond strength. Correlations among $\rho_{\text{OP}}$, $J_{\text{OP}}^{\text{intra}}$, and $\rho_{\text{OCP}}$ remain strong across all bond types, reflecting their robustness in describing intramolecular interactions. These trends suggest passive and donor bonds are governed by tightly interrelated descriptors, while hydrogen bonds involve more complex relationships.

As a final remark, the comparative results show that the OP/TOP descriptors provide a clear visualization of how molecular fragment interactions affect regions of the electron density. From the perspective of orbital overlap, this leads to a straightforward interpretation. For instance, Figure 3 shows that, for the 0.001–0.003 $e/a_{0}^{3}$ isovalues, the electron density of the O–H(D) bond overlaps with that of the X⋯H interaction. This overlap complicates direct analysis based solely on the total electron density. In this context, the OP/TOP method offers a complementary perspective. It isolates the electron density specifically associated with each chemical bond, enabling a clearer interpretation of the interactions involved. We propose that OP/TOP works in synergy with established approaches such as QTAIM, NBO, and LVM. These methods provide detailed insights into the electronic structure from different theoretical standpoints. Together, they contribute to a more complete and nuanced understanding of chemical bonding.

## 4 Conclusions

This study provides a comprehensive analysis of hydrogen bond interactions, demonstrating how the OP/TOP model offers a refined approach for capturing weak orbital interactions, particularly in hydrogen bonds. By systematically comparing OP/TOP descriptors with QTAIM and NBO analyses, we establish the advantages of this model in elucidating the role of electron‐donating and withdrawing groups in modulating bond properties.

Our findings reveal that the introduction of $-{\text{CH}}_{3}$ electron‐donating groups attached to the proton bond acceptor (X) enhances its lone pair electron density, thereby strengthening the n(X)$\to \sigma^{*}{\text{X}}^{\prime }\text{‐H}$ orbital interactions, as indicated by the increased occupation of the $\sigma^{*}$(X–H) antibonding NBO. QTAIM analysis captures this interaction by showing a less negative (less covalent) ${\text{H}}_{\text{BCP}}$ for the ${\text{X}}^{\prime }\text{‐H}$ bond and a less positive (less ionic) ${\text{H}}_{\text{BCP}}$ for the X⋯H–${\text{X}}^{\prime }$ hydrogen bond. Meanwhile, OP/TOP reflects the effect of the $-{\text{CH}}_{3}$ groups by indicating a decrease in OP density for the ${\text{X}}^{\prime }\text{‐H}$ bond and an increase in OP density for the X⋯H–${\text{X}}^{\prime }$ hydrogen bond, further supporting the strengthening of the n(X)$\to \sigma^{*}{\text{X}}^{\prime }\text{‐H}$ orbital interactions.

The nonconventional ${\left({\text{CH}}_{3}\right)}_{3}\text{N}\text{⋯}\text{H}-{\text{CX}}_{3}$ (X = F, Cl) hydrogen bonds are expected to form stronger interactions when X = Cl, due to the higher electrophilicity of H in $\text{H}-{\text{CCl}}_{3}$, as indicated by its lower ${\text{pK}}_{a}$ and weaker H–C bond dissociation energy. Upon hydrogen bond formation, the $\sigma^{*}(\text{C}-\text{H})$ NBO occupation increases for both ${\left({\text{CH}}_{3}\right)}_{3}\text{N}\text{⋯}\text{H}-{\text{CF}}_{3}$ and ${\left({\text{CH}}_{3}\right)}_{3}\text{N}\text{⋯}\text{H}-{\text{CCl}}_{3}$, following the expected trend. However, QTAIM reveals an increase in the covalent character (less negative HBCP) of the C–H (donor) bond compared to the isolated ${\text{HCX}}_{3}$ proton donors, which contradicts the increase in $\sigma^{*}(\text{C}-\text{H})$ NBO occupation. In contrast, OP/TOP provides an alternative perspective, indicating a decrease in OP density (reduced electron‐sharing) for the C–H bond upon hydrogen bond formation, along with an increase in OP density for the N⋯H–C interaction, aligning with the expected trend.

Systems involving substituted phenols as proton donors provide insight into how electron‐withdrawing ($-{\text{NO}}_{2}$) and electron‐donating ($-\text{N}{\left({\text{CH}}_{3}\right)}_{2}$) groups influence hydrogen bonding. The electron‐donating substituent in the para position of phenol increases electron density in the aromatic ring, reducing the effectiveness of the O–H bond as a proton donor. QTAIM captures this effect by indicating a more covalent O–H (donor) bond (more negative ${\text{H}}_{\text{BCP}}$) and reduced covalence in the N⋯H−O interaction (less negative ${\text{H}}_{\text{BCP}}$), along with an increase in $\sigma^{*}$(O–H) NBO occupation when the electron‐donating ($-\text{N}{\left({\text{CH}}_{3}\right)}_{2}$) group is present. OP/TOP follows the trends observed in QTAIM and NBO analyses, showing a decrease (or increase) in OP density for the N⋯H–O interaction when the substituent is electron‐donating (or electron‐withdrawing).

A key contribution of this work is to demonstrate the capability of the OP/TOP model to isolate overlap densities, offering a direct visualization of orbital interactions in hydrogen bonding. This provides an advantage over QTAIM, which relies solely on total electron density and may obscure subtle interactions, and over NBO, which focuses on individual orbital interactions.

In summary, we propose that the OP/TOP model constitutes a major advancement in hydrogen bond analysis, offering an alternative yet complementary perspective to conventional electron density‐based methods. Its capability to capture weak orbital interactions with high resolution makes it a valuable tool for computational chemistry and molecular design.

## Conflicts of Interest

The authors declare no conflicts of interest.

## Supporting information




**Data S1.** Supporting information.

## Data Availability

The data that support the findings of this study are openly available on the GPQTC webpage at http://gpqtc.chembos.website.
