# Supplementary material for: Orbital Interactions in Hydrogen Bonds: A Perspective From the Chemical Bond Overlap Model
Source: J Comput Chem. 2025 Jul 10;46(19):e70166. doi: 10.1002/jcc.70166 (PMC12242117; doi:10.1002/jcc.70166)
Supplement: Supplementary file 1 — Data S1. Supporting information. [file JCC-46-0-s001.pdf]

Supporting Information

Orbital Interactions in Hydrogen Bonds: A Perspective from the Chemical Bond Overlap Model

Rodolfo A. Santos[a], Carlos V. Santos-Jr.*[a], Eduardo C. Aguiar[b], Albano N. Carneiro Neto[c], Renaldo T. Moura Jr.*[d,e]

[a]*Department of Chemistry, Federal University of Paraiba, Joao Pessoa, PB 58051-970, Brazil*
[b]*Academic Unity of Belo Jardim, Federal Rural University of Pernambuco, Belo Jardim, PE 55156-580, Brazil*
[c]*Physics Department and CICECO-Aveiro Institute of Materials, University of Aveiro, Aveiro, 3810-193, Portugal*
[d]*Computational and Theoretical Chemistry Group (CATCO), Department of Chemistry, Southern Methodist University, Dallas, TX 75275, USA*
[e]*Academic Unit of Cabo de Santo Agostinho, Federal Rural University of Pernambuco, Cabo de Santo Agostinho, PE 54518-430, Brazil*

Contents

1 Assessment to base set effect on QTAIM and OP/TOP descriptors 2

2 Correlation Matrix and Heat maps 6

3 Cartesian coordinates of each studied chemical systems 9

Email address: `renaldotmjr@gmail.com` (Renaldo T. Moura Jr.*)

1. Assessment to base set effect on QTAIM and OP/TOP descriptors

Table S1: Results for the basis set assessment of the electron density at the bond critical point (ρ_{BCP}, in e/a_0^3) of the hydrogen bond in systems **2**–**4** and **8**–**11**. The first row presents the molecular formulas of the proton acceptors in these dimers. Calculations were performed at the ωB97X-D level of theory using the following basis set families: Pople's (6-31G, 6-31++G, and 6-311++G(2d,2p)), Ahlrichs' (def2-SV, def2-TZVP, and def2-QZVP), and Dunning's (aug-cc-pVDZ, aug-cc-pVTZ, and aug-cc-pVQZ).

Basis set	H_2O	CH_3HO-H_2O	$(CH_3)_2O$	NH_3	$(CH_3)NH_2$	$(CH_3)_2NH$	$(CH_3)_3N$
6-31g	0.0292	0.0313	0.0325	0.0327	0.0359	0.0377	0.0383
6-31++g	0.0274	0.0299	0.0316	0.0305	0.0342	0.0364	0.0375
6-311++g(2d,2p)	0.0246	0.0269	0.0284	0.0280	0.0314	0.0335	0.0346
def2-SV	0.0300	0.0318	0.0326	0.0335	0.0364	0.0378	0.0382
def2-TZVP	0.0250	0.0273	0.0288	0.0284	0.0319	0.0339	0.0349
def2-QZVP	0.0255	0.0280	0.0296	0.0290	0.0325	0.0347	0.0358
aug-cc-pVDZ	0.0248	0.0271	0.0286	0.0278	0.0312	0.0333	0.0344
aug-cc-pVTZ	0.0258	0.0283	0.0299	0.0295	0.0331	0.0353	0.0365
aug-cc-pVQZ	0.0257	0.0281	0.0297	0.0291	0.0326	0.0348	0.0359

Table S2: Results for the basis set assessment of the Laplacian $\nabla^2\rho_{\mathrm{BCP}}$ (in e/a_0^5) for hydrogen bond in systems **2**–**4** and **8**–**11**. The first row presents only the molecular formulas of the proton acceptors in these dimers. Calculations were performed at the ωB97X-D level of theory using the following basis set families: Pople's (6-31G, 6-31++G, and 6-311++G(2d,2p)), Ahlrichs' (def2-SV, def2-TZVP, and def2-QZVP), and Dunning's (aug-cc-pVDZ, aug-cc-pVTZ, and aug-cc-pVQZ).

Basis set	H_2O	CH_3HO-H_2O	$(CH_3)_2O$	NH_3	$(CH_3)NH_2$	$(CH_3)_2NH$	$(CH_3)_3N$
6-31g	0.093	0.101	0.105	0.092	0.100	0.105	0.106
6-31++g	0.095	0.102	0.105	0.094	0.101	0.106	0.106
6-311++g(2d,2p)	0.083	0.089	0.092	0.075	0.080	0.083	0.082
def2-SV	0.090	0.099	0.104	0.089	0.097	0.104	0.105
def2-TZVP	0.089	0.095	0.098	0.076	0.080	0.083	0.082
def2-QZVP	0.085	0.091	0.094	0.074	0.079	0.081	0.080
aug-cc-pVDZ	0.087	0.095	0.099	0.083	0.092	0.097	0.098
aug-cc-pVTZ	0.080	0.084	0.085	0.066	0.068	0.069	0.066
aug-cc-pVQZ	0.082	0.087	0.090	0.070	0.075	0.077	0.077

Table S3: Results for the basis set assessment of the local energy density H_{BCP} (in eV/a_0^3) of the hydrogen bond in systems **2**–**4** and **8**–**11**. The first row presents only the molecular formulas of the proton acceptors in these dimers. Calculations were performed at the ωB97X-D level of theory using the following basis set families: Pople's (6-31G, 6-31++G, and 6-311++G(2d,2p)), Ahlrichs' (def2-SV, def2-TZVP, and def2-QZVP), and Dunning's (aug-cc-pVDZ, aug-cc-pVTZ, and aug-cc-pVQZ).

Basis set	H_2O	CH_3HO-H_2O	$(CH_3)_2O$	NH_3	$(CH_3)NH_2$	$(CH_3)_2NH$	$(CH_3)_3N$
6-31g	-0.002	-0.002	-0.002	-0.002	-0.003	-0.003	-0.003
6-31++g	-0.001	-0.001	-0.001	-0.001	-0.002	-0.002	-0.002
6-311++g(2d,2p)	0.001	0.001	0.000	0.000	-0.001	-0.002	-0.002
def2-SV	-0.002	-0.001	-0.001	-0.002	-0.002	-0.002	-0.002
def2-TZVP	0.001	0.001	0.000	-0.001	-0.002	-0.003	-0.003
def2-QZVP	0.001	0.000	0.000	-0.001	-0.002	-0.003	-0.004
aug-cc-pVDZ	0.002	0.002	0.002	0.002	0.002	0.001	0.001
aug-cc-pVTZ	-0.001	-0.002	-0.002	-0.003	-0.005	-0.006	-0.007
aug-cc-pVQZ	0.000	-0.001	-0.001	-0.002	-0.003	-0.004	-0.004

Table S4: Results for the basis set assessment of the overlap density ρ_{OP} (in e) of the hydrogen bond in systems **2**–**4** and **8**–**11**. The first row presents only the molecular formulas of the proton acceptors in these dimers. Calculations were performed at the ωB97X-D level of theory using the following basis set families: Pople's (6-31G, 6-31++G, and 6-311++G(2d,2p)), Ahlrichs' (def2-SV, def2-TZVP, and def2-QZVP), and Dunning's (aug-cc-pVDZ, aug-cc-pVTZ, and aug-cc-pVQZ).

Basis set	H_2O	CH_3HO-H_2O	$(CH_3)_2O$	NH_3	$(CH_3)NH_2$	$(CH_3)_2NH$	$(CH_3)_3N$
6-31g	0.088	0.092	0.091	0.062	0.076	0.086	0.090
6-31++g	0.030	0.030	0.041	0.015	0.012	0.011	0.046
6-311++g(2d,2p)	0.0494	0.0562	0.081	0.014	0.026	0.039	0.038
def2-SV	0.120	0.121	0.118	0.121	0.134	0.140	0.140
def2-TZVP	0.038	0.064	0.088	0.011	0.058	0.106	0.161
def2-QZVP	0.061	0.066	0.063	0.038	0.060	0.090	0.108
aug-cc-pVDZ	0.052	0.103	0.193	0.046	0.129	0.284	0.370
aug-cc-pVTZ	0.021	0.028	0.023	0.058	0.144	0.219	0.301
aug-cc-pVQZ	0.028	0.046	0.101	0.063	0.121	0.229	0.205

Table S5: Results for the basis set assessment of the intra-overlap repulsion $J_{\mathrm{OP}}^{\mathrm{intra}}$ (in eV) of the hydrogen bond in systems **2**–**4** and **8**–**11**. The first row presents only the molecular formulas of the proton acceptors in these dimers. Calculations were performed at the ωB97X-D level of theory using the following basis set families: Pople's (6-31G, 6-31++G, and 6-311++G(2d,2p)), Ahlrichs' (def2-SV, def2-TZVP, and def2-QZVP), and Dunning's (aug-cc-pVDZ, aug-cc-pVTZ, and aug-cc-pVQZ).

Basis set	H_2O	CH_3HO-H_2O	$(CH_3)_2O$	NH_3	$(CH_3)NH_2$	$(CH_3)_2NH$	$(CH_3)_3N$
6-31g	0.124	0.135	0.134	0.056	0.086	0.110	0.120
6-31++g	0.017	0.017	0.031	0.003	0.002	0.003	0.041
6-311++g(2d,2p)	0.042	0.052	0.110	0.003	0.013	0.027	0.029
def2-SV	0.224	0.230	0.219	0.208	0.258	0.286	0.287
def2-TZVP	0.022	0.061	0.116	0.003	0.050	0.162	0.366
def2-QZVP	0.062	0.072	0.069	0.026	0.061	0.132	0.181
aug-cc-pVDZ	0.036	0.148	0.494	0.033	0.240	1.119	2.023
aug-cc-pVTZ	0.007	0.010	0.006	0.053	0.289	0.710	1.404
aug-cc-pVQZ	0.017	0.052	0.234	0.074	0.252	0.936	0.772

Table S6: Results for the basis set assessment of the overlap critical point density ρ_{OCP} (in e/a_0^3) of the hydrogen bond in systems **2**–**4** and **8**–**11**. The first row presents only the molecular formulas of the proton acceptors in these dimers. Calculations were performed at the ωB97X-D level of theory using the following basis set families: Pople's (6-31G, 6-31++G, and 6-311++G(2d,2p)), Ahlrichs' (def2-SV, def2-TZVP, and def2-QZVP), and Dunning's (aug-cc-pVDZ, aug-cc-pVTZ, and aug-cc-pVQZ).

Basis set	H_2O	CH_3HO-H_2O	$(CH_3)_2O$	NH_3	$(CH_3)NH_2$	$(CH_3)_2NH$	$(CH_3)_3N$
6-31g	0.0085	0.0090	0.0090	0.0039	0.0049	0.0056	0.0059
6-31++g	0.0004	0.0008	0.0016	0.0018	0.0041	0.0048	0.0103
6-311++g(2d,2p)	0.0008	0.0030	0.0063	0.0026	0.0041	0.0048	0.0062
def2-SV	0.0123	0.0128	0.0128	0.0080	0.0093	0.0103	0.0105
def2-TZVP	0.0033	0.0053	0.0071	0.0025	0.0046	0.0068	0.0091
def2-QZVP	0.0063	0.0069	0.0069	0.0036	0.0050	0.0071	0.0077
aug-cc-pVDZ	0.0029	0.0070	0.0119	0.0038	0.0106	0.0253	0.0423
aug-cc-pVTZ	0.0009	0.0019	0.0019	0.0053	0.0097	0.0205	0.0308
aug-cc-pVQZ	0.0059	0.0111	0.0241	0.0086	0.0146	0.0305	0.0298

Table S7: Results for the basis set assessment of the Laplacian of ρ_{OCP} at OCP $\nabla^2\rho_{\text{OCP}}$ (in e/a_0^5) of the hydrogen bond in systems **2**–**4** and **8**–**11**. The first row presents only the molecular formulas of the proton acceptors in these dimers. Calculations were performed at the ωB97X-D level of theory using the following basis set families: Pople's (6-31G, 6-31++G, and 6-311++G(2d,2p)), Ahlrichs' (def2-SV, def2-TZVP, and def2-QZVP), and Dunning's (aug-cc-pVDZ, aug-cc-pVTZ, and aug-cc-pVQZ).

Basis set	H_2O	CH_3HO-H_2O	$(CH_3)_2O$	NH_3	$(CH_3)NH_2$	$(CH_3)_2NH$	$(CH_3)_3N$
6-31g	-0.0750	-0.0807	-0.0806	-0.0193	-0.0255	-0.0308	-0.0315
6-31++g	-0.0423	-0.0545	-0.0773	-0.0931	-0.1497	-0.1630	-0.3193
6-311++g(2d,2p)	-0.0094	-0.0501	-0.0459	-0.0183	-0.0221	-0.0207	-0.2360
def2-SV	-0.1251	-0.1312	-0.1360	-0.0498	-0.0618	-0.0711	-0.0760
def2-TZVP	-0.0343	-0.0550	-0.0706	-0.0182	-0.0240	-0.0285	-0.0310
def2-QZVP	-0.0557	-0.0586	-0.0512	-0.0141	-0.0189	-0.0292	-0.0260
aug-cc-pVDZ	-0.0104	-0.0853	-0.2304	-0.0149	-0.2099	-0.5273	-0.9258
aug-cc-pVTZ	-0.0090	-0.0122	-0.0152	-0.0228	-0.0488	-0.1729	-0.3224
aug-cc-pVQZ	-0.0452	-0.1127	-0.3157	-0.0584	-0.1034	-0.2652	-0.2499

2. Correlation Matrix and Heat maps

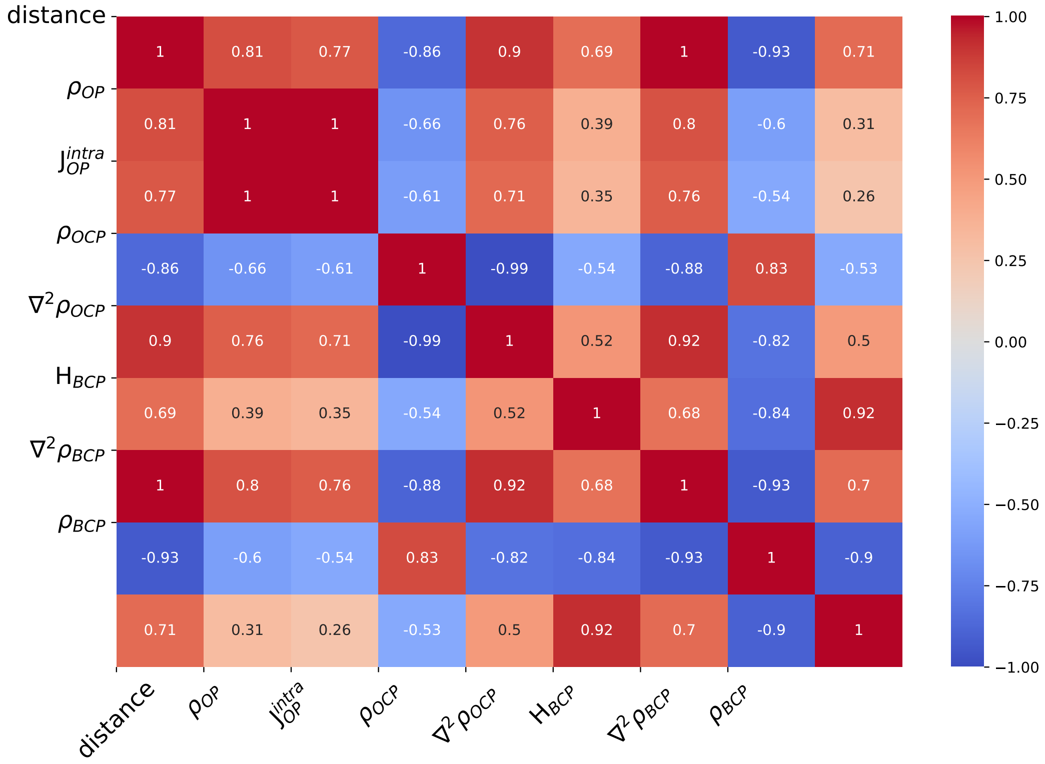

Figure S1: Correlation between all descriptors used to investigate passive bonds across the studied systems. Calculations were performed at the ωB97X-D/def2-TZVP level of theory.

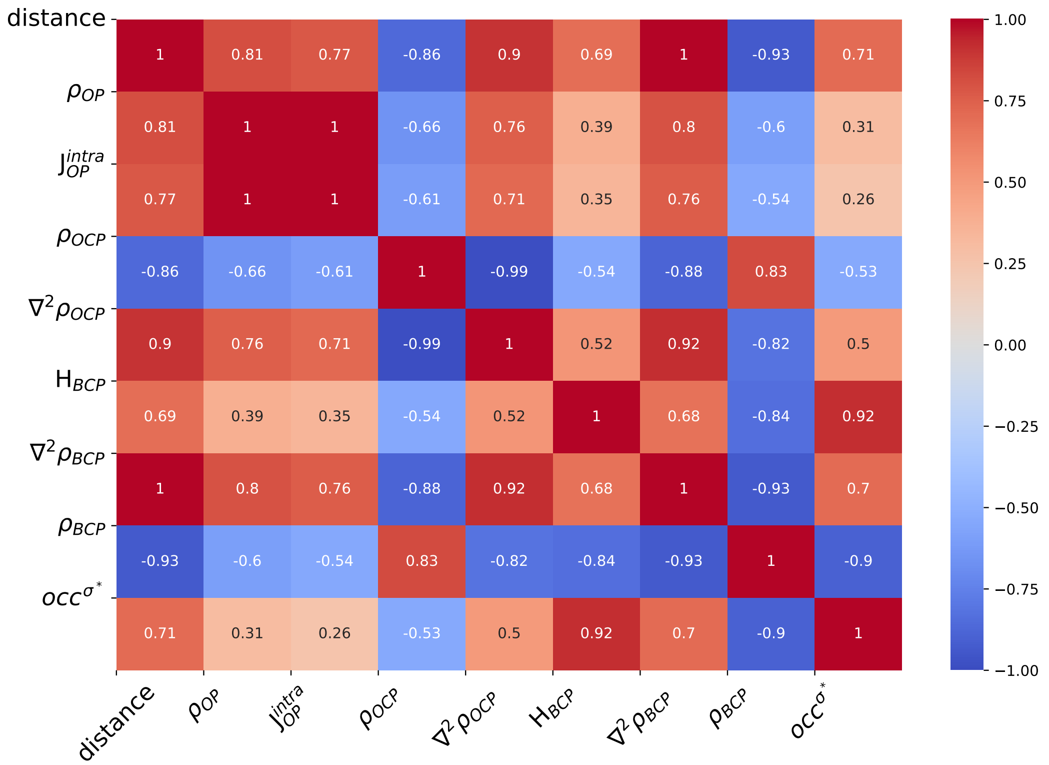

Figure S2: Correlation between all descriptors used to investigate donnor bonds across the studied systems. Calculations were performed at the ωB97X-D/def2-TZVP level of theory.

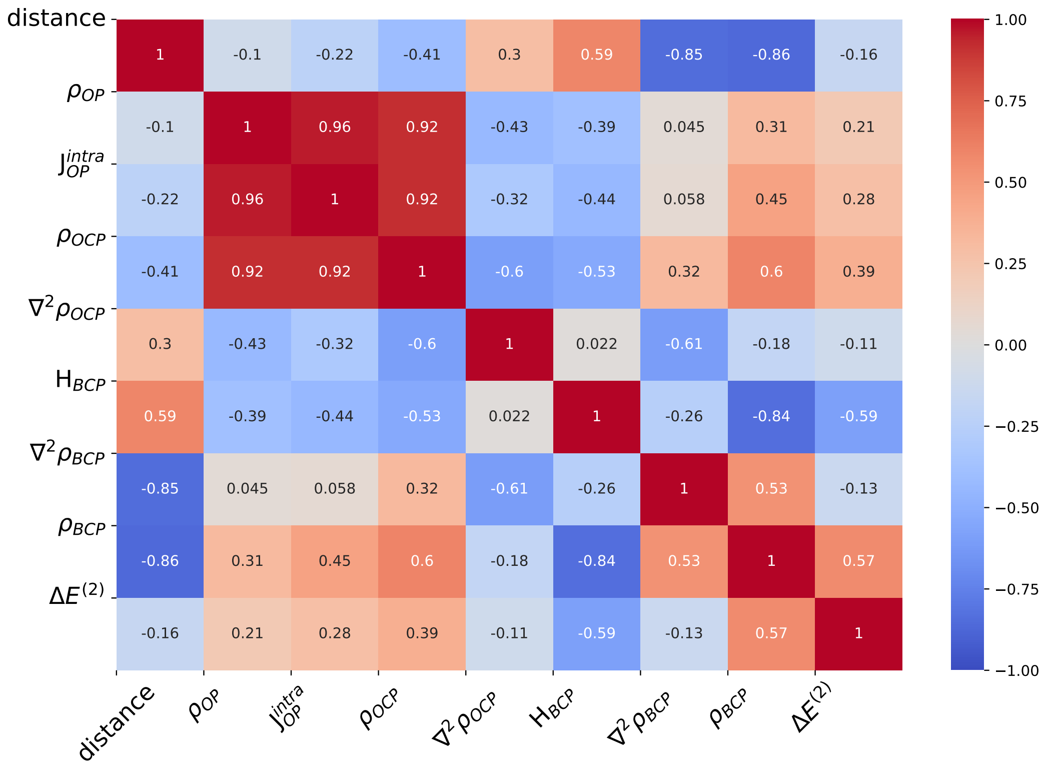

Figure S3: Correlation between all descriptors used to investigate hydrogen bonds across the studied systems. Calculations were performed at the ωB97X-D/def2-TZVP level of theory.

3. Cartesian coordinates of each studied chemical systems

H_2O

O	0.000000000000	0.000000000000	0.110811885567
H	0.000000000000	0.783975956807	-0.443247536978
H	0.000000000000	-0.783975956807	-0.443247536978

$H_2O \cdots H_2O$

O	-1.383024108149	-0.002784001507	0.109944008420
H	-1.732438131129	-0.755382059953	-0.369665027746
H	-1.728061131474	0.774373056646	-0.332319026036
O	1.509186113350	0.003364000939	-0.119558010720
H	1.897543147352	-0.021445003268	0.754523057109
H	0.553662042181	-0.002191000190	0.024374999486

$CH_3HO \cdots H_2O$

O	2.018740152306	-0.186363013542	0.107930007436
H	1.145054088835	0.216231015750	0.004303999653
H	2.502919189653	0.049494006308	-0.682861053824
O	-0.695837051481	0.699734055621	-0.102990005523
H	-0.974497075835	1.288469098015	0.599050045796
C	-1.413939108711	-0.519176037124	0.001130999951
H	-1.035112080705	-1.176524090496	-0.778967061308
H	-1.252615095684	-1.003586076215	0.968106075197
H	-2.485338191032	-0.366005026221	-0.155936009655

$(CH_3)_2O \cdots H_2O$

O	-2.300455344465	-0.000443609288	0.167823039910
H	-1.389160272900	-0.000440709397	-0.158886982459
H	-2.853122385248	0.001008453083	-0.613125017596
O	0.476989868080	-0.000002915767	-0.454812007240
C	1.001855036890	1.171780117003	0.122145036579
H	0.524596092762	2.019097235822	-0.366803999198
H	0.789434028540	1.213776147369	1.196905119404
H	2.086094128898	1.231778005948	-0.028692974710
C	1.002616782248	-1.171470061469	0.122082038028
H	0.789926761352	-1.213856042550	1.196776116574
H	0.526173649518	-2.019065072428	-0.367177000366
H	2.086950856280	-1.230586182364	-0.028445975938

$H_2CO \cdots H_2O$

O	1.828482140206	0.150610009980	-0.071796006839
H	2.453835189877	-0.250755021026	0.530881040989
H	1.057150079998	-0.430148034642	-0.072420007359
C	-1.295068097063	0.470363034309	0.020715003253
H	-0.574058041513	1.305252097345	0.044103002501
H	-2.369538180171	0.725565054556	0.026595003881
O	-0.928105069518	-0.672122052444	-0.009884999261

$CH_3HCO\cdots H_2O$

O	-2.519090237829	-0.334933743741	-0.071959004015
H	-3.164975269509	0.002434310597	0.547950039454
H	-1.797515153271	0.309120276448	-0.070817007761
C	0.664732012785	-0.160017865991	0.016903000855
H	0.101318932563	-1.111695913956	0.053860004368
O	0.067578014364	0.886462243798	-0.011483003425
C	2.153831123983	-0.276655934860	0.004761002417
H	2.479089126315	-0.817875993314	0.897071067576
H	2.458824119519	-0.877696994180	-0.855908062743
H	2.623977200911	0.703529119055	-0.034604001001

$(CH_3)_2CO\cdots H_2O$

O	2.765433211791	-0.031461000356	-0.028886002696
H	3.331109255016	-0.576439042794	0.517260039766
H	1.919698147734	-0.501582036617	-0.077884005877
C	-0.732115057083	-0.089757007643	-0.011221002478
O	0.070280003981	-0.994555073783	-0.062849006003
C	-2.211540167865	-0.366688030004	0.040025003867
H	-2.587106199284	-0.104497007076	1.032660079712
H	-2.745514207539	0.258875018366	-0.677775051907
H	-2.407374184138	-1.418596106632	-0.152988010827
C	-0.311400024954	1.355536101785	-0.002748001579
H	-0.526013038931	1.782527133753	-0.986457074341
H	-0.894156068241	1.923058148093	0.724825053091
H	0.753981057895	1.450238112067	0.197896013634

$NH_3\cdots H_2O$

O	1.552613414713	-0.103944101519	-0.000423950353
H	0.586922345728	0.007963964136	0.003309051173
H	1.913640491720	0.781839943425	0.002567049418
N	-1.383784805736	0.019969085586	0.000457050390
H	-1.628542872670	-0.825678965855	-0.498208986747
H	-1.860498792549	0.790462172309	-0.448704984281
H	-1.745934839199	-0.062817900964	0.941230120531

$(CH_3)NH_2\cdots H_2O$

O	-2.035467154099	-0.187774011759	-0.091298008834
H	-1.152737086311	0.211891016168	0.015018002701
H	-2.529599195275	0.065796004686	0.687452052759
N	0.710467056131	0.711151054768	0.001111002343
H	1.066698083311	1.320501101790	0.724639057872
H	0.852229063755	1.183093090486	-0.881928067917
C	1.399433106166	-0.577305046654	0.016758000997
H	1.225036092000	-1.064570081672	0.976100075433
H	0.971505075919	-1.218851094773	-0.753349056215
H	2.480737190647	-0.509892040340	-0.145870010608

$(CH_3)_2NH\cdots H_2O$

O	-2.254206173670	0.025021003196	-0.135927008229
H	-1.354111101538	-0.019209001844	0.239605018260
H	-2.792164214224	-0.544879043958	0.412452029930
N	0.533711038582	-0.008330001331	0.501802036767
H	0.899183066894	-0.001059000094	1.443669112077
C	1.004171076372	-1.188918088431	-0.203667014332
H	0.759453057572	-2.083753159875	0.369257026795
H	0.490903038645	-1.257450098248	-1.164624090107
H	2.086796161895	-1.178227088719	-0.393583029759
C	0.884230068327	1.231492091480	-0.173113013197
H	0.370857025958	1.270643095619	-1.135458089145
H	0.543174040154	2.080649159594	0.418831034120
H	1.963177149715	1.335976101963	-0.354667027945

$(CH_3)_3N\cdots H_2O$

O	-2.514971106247	-0.049728892214	0.002109909068
H	-1.541722047328	-0.133349036592	0.000114910840
H	-2.851667264233	-0.944635910597	-0.027879094810
N	0.355235115597	-0.024869303954	0.000177909391
C	0.531044334504	1.416460781181	-0.021194093875
H	0.047877359229	1.831796885327	-0.905955159821
H	0.061586362628	1.857480884115	0.858442975227
H	1.594518458343	1.704606651688	-0.034191094577
C	0.900496072068	-0.608796427076	1.210598004063
H	0.416200097132	-0.166379322664	2.081771068280
H	0.709375903227	-1.682865478498	1.221143003772
H	1.987721178997	-0.450122570754	1.298625009568
C	0.910844069671	-0.643465432210	-1.188189183813
H	0.727513897946	-1.718786485253	-1.167828178973
H	0.430002092849	-0.231513331903	-2.076167250550
H	1.997410176297	-0.479509572010	-1.273489190785

$HCN\cdots H_2O$

O	2.211431088558	-0.096125539416	-0.020682167804
H	1.248821831452	-0.095850457206	-0.024814523525
H	2.458169537846	0.828301409548	-0.002745461528
C	-2.020366562705	0.014241006454	-0.036855967702
H	-3.082484749220	0.039330014219	-0.147116217738
N	-0.884822275878	-0.012603239911	0.080181333651

$CH_3CN\cdots H_2O$

O	3.075654039330	0.168020423024	0.000165907651
H	2.113169969686	0.109690368473	-0.000724094397
H	3.375423108192	-0.740651628045	0.001983906674
C	-1.088927269052	-0.062655813973	-0.000211094096
N	0.050574824381	-0.174437760814	-0.000773096210
C	-2.534677386536	0.085252121722	0.000411906279
H	-2.954852393282	-0.382570935876	0.889885972974
H	-2.795212460391	1.143102192093	-0.002828092555
H	-2.956156391859	-0.388246933383	-0.885438158819

$CH_3CH_2CN\cdots H_2O$

O	-3.695310416539	0.361181527970	0.063272929897
H	-2.742941331880	0.229885614436	-0.013437075086
H	-4.070361357407	-0.512653578273	-0.043089077725
C	0.400525972852	-0.428913114864	-0.037415075216
N	-0.733322126263	-0.291451222732	-0.125453082316
C	1.847034099703	-0.580334983288	0.079277932835
H	2.177204195140	-1.257885997423	-0.710338127386
H	2.052118166040	-1.073374998417	1.031369003644
C	2.581227018880	0.758554197097	-0.008953075791
H	2.254237924305	1.434638215479	0.780092986449
H	3.652671118309	0.594216292090	0.099048931502
H	2.400086958327	1.240044214962	-0.969117147707

HCF_3

C	-0.000001534614	-0.000096310524	0.336706084822
H	0.000015928235	-0.000170437091	1.427677354445
F	1.009193296555	-0.735696877961	-0.127720133744
F	-1.141872003159	-0.505934222622	-0.127712229423
F	0.132678018673	1.241714245442	-0.127669177150

$(CH_3)_3N\cdots HCF_3$

C	1.636376124734	0.000239997758	0.145160013493
H	0.591952043185	0.011312999942	0.472104038041
F	2.426705187797	0.658937050941	0.996581076506
F	2.090092158266	-1.252673093510	0.040153001260
F	1.747752134416	0.581073042672	-1.057235081739
N	-1.620344123172	-0.000221000294	0.110063009800
C	-1.648472127085	-0.961413073102	-0.974624073083
H	-0.913099068557	-0.686585054046	-1.732689130482
H	-1.394809107738	-1.953606148358	-0.598158043458
H	-2.637214199371	-1.019144077840	-1.460267111040
C	-2.533322195352	-0.371069030239	1.171581088148
H	-2.281163171832	-1.363394103469	1.548466120319
H	-2.451885187590	0.338403024494	1.996387154964
H	-3.584210272249	-0.388108029647	0.835455065730
C	-1.878761143921	1.342978101237	-0.369140030997
H	-1.801298135588	2.052716157498	0.455962036405
H	-1.139705085825	1.615225121762	-1.124323083262
H	-2.882031221942	1.444281110857	-0.816728064983

$HCCl_3$

C	-0.000953471567	-0.000158943255	0.454559176934
H	0.000092553101	-0.000303193057	1.537173721246
Cl	-0.783905343395	-1.486700456563	-0.083670791403
Cl	-0.896342385212	1.421880508730	-0.083634781950
Cl	1.680578834812	0.064893882892	-0.083549060527

$(CH_3)_3N\cdots HCCl_3$

C	-1.012706282057	0.000680998054	0.049787207811
H	0.065824659105	-0.001308943098	0.224152566330
Cl	-1.833919891532	-0.371282201356	1.569304596499
Cl	-1.458763390386	1.609637417479	-0.536400772454
Cl	-1.353632042666	-1.233075289266	-1.173773617660
N	2.134887911995	-0.000039531003	0.112484722804
C	2.358767301154	0.374709545870	-1.270660764848
H	1.856365701272	-0.333873238667	-1.930870243650
H	1.942243141166	1.365680509025	-1.456896045585
H	3.429841442184	0.393399398904	-1.531985021901
C	2.718110737241	0.961727414971	1.026389927282
H	2.301180454882	1.951837214650	0.836530142229
H	2.484227294524	0.684327710904	2.055203962623
H	3.814703736715	1.023693398774	0.926450289926
C	2.601461603276	-1.345914907966	0.381327402150
H	2.368000803844	-1.620069178549	1.411135353018
H	2.097452897380	-2.050492676409	-0.281871272459
H	3.689504839399	-1.449894474945	0.234481196045

HOC_6H_5

C	-0.935650517951	-0.025885926106	-0.000106490254
H	-2.669739279923	0.769726504684	-0.000362283370
O	-2.290898958984	-0.110102677456	-0.000259786182
C	-0.218438177013	-1.216111237232	0.000117640495
C	1.164811889821	-1.181220152282	0.000274756818
C	1.845338224206	0.029141754166	0.000212804503
C	1.123134842209	1.211346795162	-0.000010491468
C	-0.263463170327	1.190237406690	-0.000171655363
H	-0.757951474794	-2.154066295644	0.000165793455
H	1.717653998314	-2.112362868546	0.000449009012
H	2.926973081510	0.049082524271	0.000337358116
H	1.638757057222	2.163534329691	-0.000061904425
H	-0.822900044450	2.119855341045	-0.000349071614

$NH_3 \cdots HOC_6H_5$

C	-0.097541587571	-0.532681217321	0.000000061739
H	-2.026541383854	-0.571615078868	0.000000078858
O	-1.268870023745	-1.193578679466	0.000000053672
C	1.076721891056	-1.283242932920	-0.000000121103
C	2.305958914857	-0.649119479445	0.000000039277
C	2.391863755549	0.737094911336	0.000000093840
C	1.223678926985	1.482303517510	-0.000000100405
C	-0.014923547102	0.859139340736	-0.000000040321
H	1.002183878216	-2.363001893919	-0.000000312989
H	3.209844520666	-1.246145607476	-0.000000025587
H	3.356154436321	1.227740166576	0.000000452588
H	1.271904283757	2.564602322924	-0.000000243319
H	-0.923538899580	1.449006293266	-0.000000097475
N	-3.608315740595	0.429421944718	0.000000030794
H	-3.710460808804	1.016527836104	-0.817759780698
H	-3.710461941243	1.016526089819	0.817760907846
H	-4.374463864053	-0.231929127635	-0.000001223821

$(CH_3)_3N \cdots HOC_6H_5$

C	1.105220377252	-0.582372035232	-0.000202162860
H	-0.828685618690	-0.672599423240	-0.000401844668
O	-0.047066663388	-1.275365515723	-0.000362032552
C	2.301125917875	-1.297698827672	0.000026634457
C	3.510988270310	-0.627109796427	0.000192166547
C	3.555568648899	0.760990216937	0.000134645053
C	2.365718975671	1.471194647691	-0.000094492652
C	1.146508410793	0.811052369107	-0.000264468011
H	2.259131464457	-2.379224605669	0.000074350327

H	4.432285654440	-1.196893403419	0.000371257169
H	4.504855387210	1.280042865801	0.000264317566
H	2.381313575194	2.554422847108	-0.000146234045
H	0.221237652192	1.374181349939	-0.000452853430
N	-2.474392276055	0.081219686308	0.000033613477
C	-2.543334033107	1.531510550996	0.000847157604
H	-2.041636037879	1.925358915520	0.885885350637
H	-2.041975362207	1.926336427610	-0.883947752264
H	-3.582136151438	1.896278429602	0.001239838921
C	-3.075628855785	-0.475899579852	-1.201072247362
H	-2.571225584749	-0.078449768324	-2.082339917947
H	-2.957013579626	-1.559712385718	-1.201327157334
H	-4.148264704713	-0.239429898691	-1.274469322302
C	-3.075357514859	-0.477221191564	1.200665077219
H	-2.956737777736	-1.561033376177	1.199703662701
H	-2.570754553497	-0.080736878744	2.082253386883
H	-4.147976313695	-0.240832839126	1.274560028574

$HOC_6H_4N(CH_3)_2$

C	-2.195805701503	-0.016877243628	0.015951122376
H	-3.920527708661	0.801833058950	0.080808679101
O	-3.557994355074	-0.084367668627	0.068135859790
C	-1.483590063821	-1.205258595037	-0.009384129396
C	-0.102001454796	-1.198170117617	-0.060842605592
C	0.625777656204	-0.000018417644	-0.098664372574
C	-0.110500782263	1.188706655661	-0.054260227472
C	-1.496031232616	1.177737218756	-0.003077829238
H	-2.023176282227	-2.143207335073	0.016490516450
H	0.408608181645	-2.149853526148	-0.072453860945
H	0.388618087722	2.146413445396	-0.060000564466
H	-2.030068392553	2.121977923908	0.027822008438
N	2.015517182395	0.005088841535	-0.191319111440
C	2.710640765359	1.238418122069	0.085554431689
H	3.781377534726	1.076697157311	-0.023204169845
H	2.427830239796	2.015235617765	-0.627880664801
H	2.520269408784	1.621283158784	1.099022896733
C	2.722569478906	-1.218826048814	0.099848871286
H	2.530802999432	-1.594740686329	1.115389798700
H	2.453264668010	-2.004950004868	-0.608337461065
H	3.791983410986	-1.045638781465	-0.004261837350

$NH_3 \cdots HOC_6H_4N(CH_3)_2$

C	1.493764041486	-0.690417694356	-0.021536425199
H	3.381135825308	-0.294160321845	-0.069569999987

O	2.789249273099	-1.072563907281	-0.081553884495
C	0.512235879620	-1.672832071478	0.010173543898
C	-0.828744399699	-1.340931756940	0.068585617589
C	-1.254327253965	-0.006449951513	0.108365466865
C	-0.258136949456	0.972796961629	0.057833120601
C	1.086354700315	0.637054354587	-0.001276462992
H	0.814163306902	-2.712172982208	-0.016638296605
H	-1.548835168532	-2.146082868369	0.085250226209
H	-0.517735563734	2.021475247251	0.064763729101
H	1.827586465378	1.426621603499	-0.039563712703
N	4.694107592112	1.065730646756	0.000558918788
H	4.682232113796	1.695918609031	-0.790769777752
H	4.626835484532	1.622812623912	0.842490583091
H	5.593247890901	0.601467258676	0.010514327114
N	-2.608572665981	0.327419320441	0.211937759982
C	-2.989751881567	1.684489581685	-0.094816059621
H	-4.068026431589	1.785870110480	0.016252803521
H	-2.527102510556	2.386805307891	0.601495500866
H	-2.716941991077	1.989498293786	-1.116542225038
C	-3.578557643209	-0.692735204951	-0.106940775129
H	-3.474301930375	-1.082604648697	-1.130581202202
H	-3.502494181697	-1.534378882189	0.584006716672
H	-4.579520979392	-0.278453221356	0.001517495789

$HOC_6H_4NO_2$

C	2.065595924272	0.016506985716	0.000000000000
H	3.790326186595	-0.796574799255	0.000000148170
O	3.411838562293	0.084074307048	0.000000178015
C	1.365766738458	1.220783877632	0.000000000000
C	-0.012429156471	1.212891288924	-0.000000172723
C	-0.685209553039	0.000670044444	-0.000000354972
C	-0.003433831169	-1.202949540695	-0.000000336398
C	1.377420703177	-1.194388040193	-0.000000153832
H	1.918238740383	2.150253107889	0.000000102713
H	-0.575182807756	2.134899109386	-0.000000176692
H	-0.556195928062	-2.130936693646	-0.000000479382
H	1.922777440730	-2.131110396078	-0.000000127426
N	-2.148263306181	-0.007352192796	-0.000000572993
O	-2.718341088098	1.063782628884	0.000000943999
O	-2.707045588047	-1.084876025620	0.000000209448

$NH_3 \cdots HOC_6H_4NO_2$

C	-1.366846244170	0.661512112103	0.000000186926
H	-3.257388477061	0.268658020829	0.000000083538

O	-2.644424673314	1.041073913303	0.000000207915
C	-0.388495462007	1.660090806265	0.000000229139
C	0.947037697961	1.325363902035	0.000000108521
C	1.314031084405	-0.012704437122	0.000000036610
C	0.361672354686	-1.017453758704	0.000000098673
C	-0.976395356338	-0.681653619512	0.000000160385
H	-0.702686182031	2.694913215344	0.000000383770
H	1.713446218959	2.086674911512	0.000000093187
H	0.678442905346	-2.050267352736	0.000000113062
H	-1.729606688882	-1.458676732320	0.000000268368
N	-4.561595153000	-1.006819947177	-0.000000391874
H	-5.443646572134	-0.509507443841	-0.000000854229
H	-4.550793724580	-1.603936665061	-0.817304423675
H	-4.550794518346	-1.603936606851	0.817303688119
N	2.729075104628	-0.369157944705	-0.000000112297
O	3.542125043766	0.533079603121	-0.000000806078
O	3.017879996607	-1.549529290330	0.000000504901
